# Priming Effect of Seeds with Niobium (Nb) on the Performance of Maize Plants Under Water Deficit Conditions

**DOI:** 10.3390/plants14203173

**Published:** 2025-10-15

**Authors:** Maisa Natália Leite Evangelista, Pedro Antônio Namorato Benevenute, Jucelino de Sousa Lima, Leônidas Canuto dos Santos, Everton Geraldo de Morais, Vitor L. Nascimento, Guilherme Lopes, Luiz Roberto Guimarães Guilherme

**Affiliations:** 1 Department of Soil Science, Federal University of Lavras, University Campus, P.O. Box 3037, Lavras 37203-202, Minas Gerais, Brazil; maisanatalia1@gmail.com (M.N.L.E.); benevenutepedro@gmail.com (P.A.N.B.); leonidas.santos2@estudante.ufla.br (L.C.d.S.); evertonmoraislp@gmail.com (E.G.d.M.); guilherme.lopes@ufla.br (G.L.); 2 Department of Biology, Institute of Natural Sciences, Federal University of Lavras, University Campus, P.O. Box 3037, Lavras 37203-202, Minas Gerais, Brazil; sousajucelino12@gmail.com (J.d.S.L.); vitor.nascimento@ufla.br (V.L.N.)

**Keywords:** biostimulant, hydropriming, oxidative stress, toxicology, *Zea mays* L.

## Abstract

Water deficit is a limitation to maize (*Zea mays* L.) productivity, and seed physiological conditioning (priming) is a strategy to mitigate its effects. Niobium (Nb), an abundant element in the Earth’s crust and crucial for emerging technologies, is primarily produced and exported by Brazil, particularly in the state of Minas Gerais. However, its behavior in soil and effects on plants remain poorly understood. This study evaluated the impact of maize seed hydropriming with different solutions, including ammonium niobate (V) oxalate (C_4_H_4_NNbO_9_), on germination, seedling physiological performance under water deficit, and recovery after rehydration. The experiment was conducted in a greenhouse using a randomized block design with eight treatments and five replications. The germination speed index (GSI) was recorded over a period of seven days. Water deficit was imposed on day eight by suspending irrigation for seven days, followed by rehydration until day 21. Morphological, biochemical, and physiological traits were evaluated, including biomass, pigments, oxidative stress, antioxidant activity, starch, and osmolytes, with photosynthetic parameters measured during rehydration. Hydropriming with Nb-enhanced seed germination but also induced oxidative stress and reduced biomass accumulation. Nb seed priming affected photosynthetic performance in a treatment-dependent manner, leading to phototoxic effects. Overall, although Nb shows biostimulant potential by improving maize germination, its use under water-deficit conditions may trigger toxic responses associated with increased oxidative stress and growth inhibition. These findings highlight the need for further studies to define safe and effective Nb concentrations for improving drought tolerance.

## 1. Introduction

Maize (*Zea mays* L.), a member of the *Poaceae* family, is one of the most widely cultivated cereals worldwide due to its high yield potential and broad versatility of use [1]. Its origin is attributed to the region of Mexico, Central America, or the southwestern United States [2]. Beyond its importance in human nutrition, maize is extensively used for animal feed and biofuel production, playing a strategic role in several production chains [3,4].

The species exhibits a C4 photosynthetic metabolism, characterized by a functional anatomy that concentrates CO_2_ in the bundle sheath cells. This adaptation minimizes photorespiration and enhances photosynthetic efficiency, leading to improved water-use efficiency compared to C3 species [5]. However, under water deficit conditions, maize growth and development are impaired due to stomatal closure, which restricts CO_2_ diffusion, reduces carbon assimilation, and consequently lowers photosynthetic rate [6].

Maize cultivation is feasible across a wide range of climates, from temperate to tropical, due to its high adaptability. According to CONAB [7], Brazilian maize production for the 2024/2025 season is estimated at 124.75 million tons, representing a 7.8% increase compared with the previous harvest. Nonetheless, successful cultivation depends on selecting varieties whose growth cycles align with the duration of the growing season and production goals [8,9]. Despite its high productive potential, maize remains sensitive to biotic and abiotic stresses, partly due to its low leaf plasticity, reduced prolificacy, and limited compensatory capacity [10], requiring careful and strategic management to sustain yields.

Water deficit is one of the major limiting factors in global agriculture, occurring when water loss exceeds root uptake capacity, leading to reduced cell turgor and, in critical cases, plant death [11]. In response, plants activate multiple defense mechanisms, including physiological adjustments, changes in membrane fluidity, osmolyte accumulation, production of secondary metabolites, and activation of antioxidant systems to scavenge reactive oxygen species (ROS) [12]. Developing strategies to mitigate these effects is thus essential for plant survival under limited water availability.

Although Brazil is rich in water resources, its uneven distribution leads to scarcity in certain regions, particularly in the Northeast, underscoring the need for alternative solutions [13]. In this context, seed physiological conditioning, or priming, emerges as a promising technique to alleviate water deficit. This practice consists of controlled seed hydration, sufficient to trigger the initial metabolic processes of germination without allowing radicle protrusion [14]. Typically, seeds are soaked in an osmotic solution for a set period, followed by drying to restore their original moisture content [15]. Esper Neto [16], in a study on seed priming with ZnO nanoparticles, reported benefits for seed germination and vigor, as well as improved seedling development, including increased root length and greater biomass production.

Niobium (Nb), a relatively abundant element in the Earth’s crust, is classified as a trace element, a concept associated with its abundance, encompassing metals and metalloids with diverse chemical behaviors [17]. Niobium can be released into the environment through natural rock weathering as well as anthropogenic activities, such as agriculture and mining [17]. Its discovery in Brazil occurred in 1953 by geologist Djalma Guimarães, with its commercial exploitation established in 1955 through the founding of the Brazilian Metallurgy and Mining Company (CBMM) in Araxá, Minas Gerais, which hosts the world’s largest Nb reserve [18]. In the Earth’s crust, Nb belongs to Group 5 (formerly Vb) of the Periodic Table, with ore contents ranging from 0.3% to 1% Nb_2_O_5_, reaching up to 2.34% in Brazilian deposits, particularly in Araxá [19,20,21].

Industrially, Nb has broad and strategic applications across multiple sectors: in mobility, it is used in lightweight, high-strength alloys; in construction, it reinforces urban structures; in energy, it enables electrification and energy storage technologies, such as batteries; and in healthcare, it is employed in high-precision medical equipment [22,23,24].

Although Nb is not considered a nutrient, recent studies suggest its potential role in physiological processes, indicating a correlation between CO_2_ sequestration and Nb accumulation [25]. This condition implies that elevated CO_2_ levels may increase its uptake. Niobium also shows low toxicity to aquatic organisms, highlighting its potential for environmentally safe application [26,27]. However, research on the use of Nb in agroecosystems remains scarce, and its potential benefits have not yet been reported. In contrast, transition metals have been increasingly studied for their priming effects aimed at enhancing maize tolerance to abiotic stresses [28]. Studies, primarily in maize, indicate that nanoparticulate forms of copper (Cu) [29] and iron (Fe) [30] can act as mild stress inducers, triggering reactive oxygen species (ROS)-mediated stress memory and activating antioxidant defenses, osmoprotectants, and stress-responsive genes. These priming effects have been shown to improve germination, growth, photosynthetic efficiency, water relations, and yield under various stress conditions. Collectively, these studies demonstrate that transition metals can modulate physiologic and biochemical pathways. Therefore, the scarcity of research on Nb highlights the need to investigate whether Nb may exert similar stress-mitigating effects, providing novel insights for agricultural applications. Nevertheless, elements such as selenium and iodine, which, although not classified as nutrients, have been demonstrated in multiple studies to alleviate various abiotic stresses, including water deficit [31,32,33]. Selenium has been shown to enhance antioxidant enzyme activities and reduce oxidative damage in plants under drought, while iodine can improve photosynthetic efficiency and stress tolerance [31,32,33]. By drawing parallels with these elements, which act through modulation of physiological and biochemical pathways, we hypothesize that niobium could also contribute to mitigating water stress, potentially through similar mechanisms.

This study investigates the use of Nb applied via seed hydropriming as a potential strategy to mitigate water stress in maize. Some evidence suggests that transition metals, such as Nb, when used at low concentrations, can influence physiological and enzymatic processes in plants, including during germination [34]. Considering Nb’s abundance but limited agricultural use, particularly as a biostimulant in economically important crops, although niobium is a strategic element for emerging technologies and abundant in Brazil, its agrarian application, particularly as a seed priming agent to enhance drought tolerance in maize, remains unexplored, representing a significant gap in the scientific literature. This research aims to advance knowledge in a field that is scarcely studied, with potential technological and economic implications. We hypothesize that morphophysiological assays will demonstrate that Nb seed priming can alleviate water stress, improve physiological responses, and thus enhance plant growth and productivity. To address this hypothesis, the study seeks to answer the following scientific questions: (i) Can Nb seed priming improve the germination and growth performance of maize under drought stress? (ii) Does Nb alleviate drought stress by regulating the antioxidant system and osmotic adjustment substances? The study aims to investigate the beneficial or detrimental effects of Nb and assess its potential as an abiotic stress mitigator, focusing on its mechanisms during germination, photosynthetic efficiency, and biochemical responses under water deficit and post-rehydration conditions.

## 2. Results

### 2.1. Germination Speed Index

**Figure 1:**
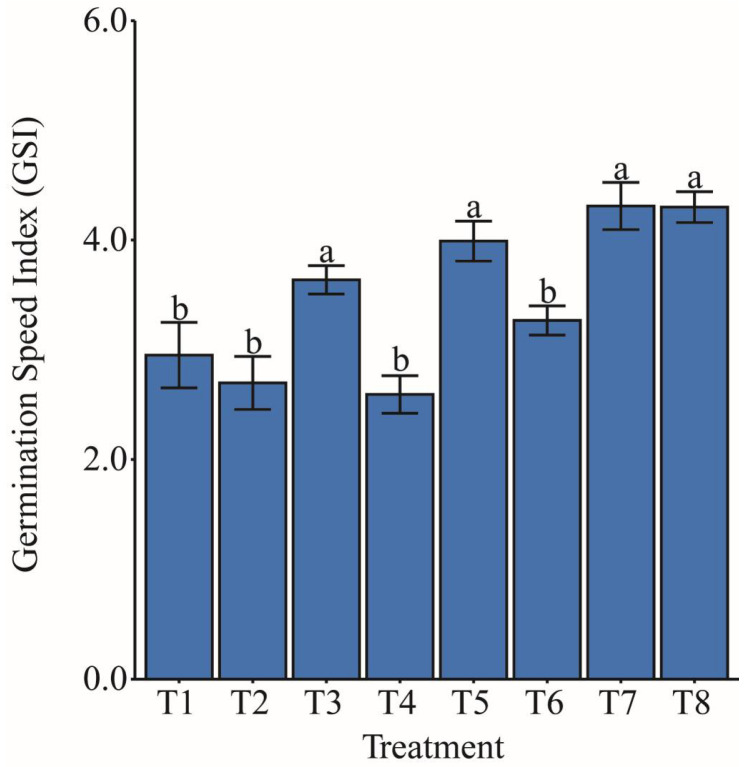
Effect of different hydropriming treatments on the germination speed index (GSI) of maize seeds. Lowercase letters indicate statistically significant differences among hydropriming treatments, as determined by the Scott–Knott test (*p* < 0.05). Bars represent the mean, and whiskers represent the associated standard error. T1 consisted of maize seed priming with distilled water (H_2_O), T2 with phosphate buffer. T3 involved priming with distilled water, T4 with phosphate buffer, T5 with ammonium oxalate, T6 with a combination of ammonium oxalate and phosphate buffer, T7 with ammonium niobate (V) oxalate, and T8 with a combination of ammonium niobate (V) oxalate and phosphate buffer.

The germination speed index (GSI) (Figure 1) showed significantly higher values in treatments T3: distilled water, T5: ammonium oxalate, T7: ammonium niobate (V) oxalate, and T8: ammonium niobate (V) oxalate + phosphate buffer, with increases of 26.44%, 38.72%, 49.84%, and 49.50%, respectively, compared with the average of treatments T1: distilled water, T2: phosphate buffer, T4: phosphate buffer, and T6: ammonium oxalate + phosphate buffer. These results indicate a positive effect of ammonium oxalate, especially when combined with niobium, on promoting germination.

### 2.2. Biomass

**Figure 2:**
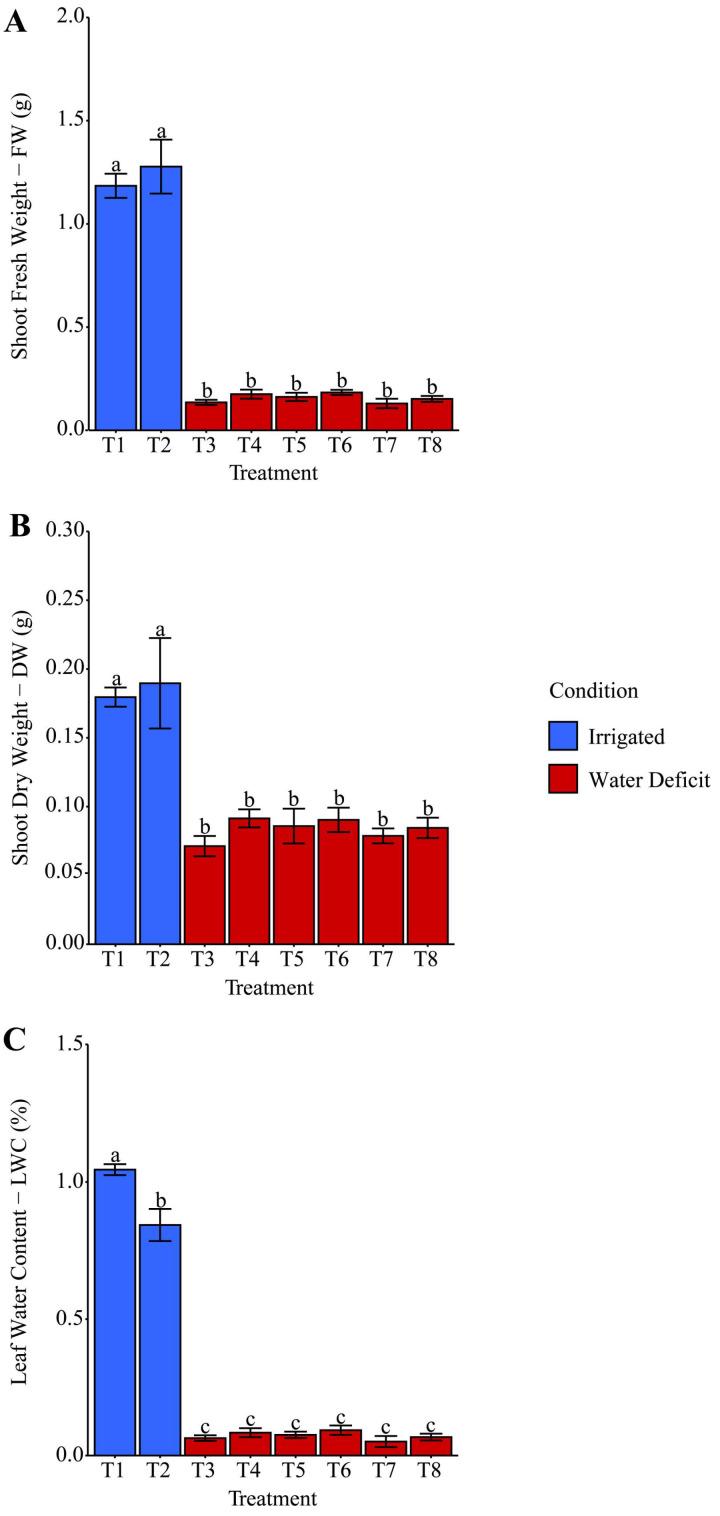
Effect of different hydropriming treatments on (**A**) shoot fresh weight (FW), (**B**) shoot dry weight (DW), and (**C**) leaf relative water content (LWC) of maize seedlings under water deficit. Lowercase letters indicate statistically significant differences among the various hydropriming treatments, as determined by the Scott–Knott test (*p* < 0.05). Bars represent the mean, and whiskers represent the associated standard error. Under irrigation conditions, treatment T1 consisted of hydropriming maize seeds with distilled water (H_2_O), while treatment T2 used a phosphate-buffer solution. Under water deficit conditions, treatments involved hydropriming with distilled water (T3), phosphate buffer (T4), ammonium oxalate (T5), a combination of ammonium oxalate and phosphate buffer (T6), ammonium niobate (V) oxalate (T7), and a combination of ammonium niobate (V) oxalate and phosphate buffer (T8).

The effects of water deficit were evaluated based on shoot fresh weight (FW), shoot dry weight (DW), and leaf relative water content (LWC), as shown in Figure 2A, Figure 2B and Figure 2C, respectively. For FW, regardless of the type of hydropriming used, water deficit resulted in an 87.30% reduction compared with irrigated treatments. Similarly, irrigation suspension reduced DW by 54.65% compared with the average of treatments T1 (distilled water) and T2 (phosphate buffer). Moreover, regardless of the priming solution tested, the water deficit condition imposed on the plants resulted in reductions of 93.05% and 91.40% in LWC values compared with treatments T1 and T2, respectively, suggesting that water deficit negatively affected the plant’s water status, irrespective of the treatment.

**Figure 3:**
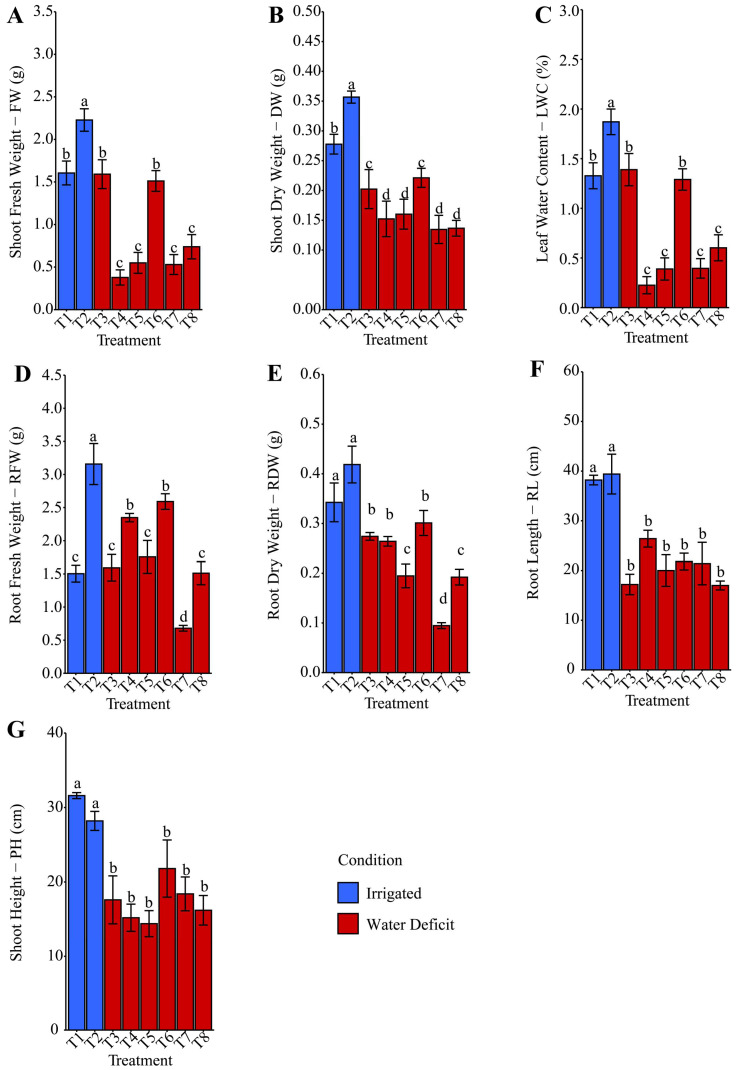
Effect of different hydropriming treatments on (**A**): shoot fresh weight (FW), (**B**) dry weight (DW), (**C**) leaf relative water content (LWC), (**D**) root fresh weight (RFW), (**E**) dry weight (RDW), (**F**) root length (RL), (**G**) shoot height (PH) of maize after rehydration. Lowercase letters indicate statistically significant differences among the various hydropriming treatments, as determined by the Scott–Knott test (*p* < 0.05). Bars represent the mean, and whiskers represent the associated standard error. Under irrigation conditions, treatment T1 consisted of hydropriming maize seeds with distilled water (H_2_O), while treatment T2 used a phosphate-buffer solution. Under water deficit conditions, treatments involved hydropriming with distilled water (T3), phosphate buffer (T4), ammonium oxalate (T5), a combination of ammonium oxalate and phosphate buffer (T6), ammonium niobate (V) oxalate (T7), and a combination of ammonium niobate (V) oxalate and phosphate buffer (T8).

After rehydration, the variables fresh weight of shoots (FW), dry weight of shoots (DW), leaf relative water content (LWC), fresh weight of roots (RFW), dry weight of roots (RDW), root length (RL), and plant height (PH) were evaluated, as shown in Figure 3A, Figure 3B, Figure 3C, Figure 3D, Figure 3E, Figure 3F and Figure 3G, respectively. Treatments T4: phosphate buffer, T5: ammonium oxalate, T7: ammonium niobate (V) oxalate, and T8: ammonium niobate (V) oxalate + phosphate buffer showed average reductions of 65.02%, 37.57%, and 69.83% in FW, DW, and LWC (Figure 3A, Figure 3B and Figure 3C), respectively, compared with treatments T1: distilled water, T3: distilled water, and T6: ammonium oxalate + phosphate buffer, which in turn reduced these same variables on average by 29.55%, 34.47%, and 28.61% relative to treatment T2.

In the root biomass (Figure 3D,E), treatment T7: ammonium niobate (V) oxalate stood out, showing the most significant reductions: 54.99% and 73.75% for RFW, and 50.78% and 68.57% for RDW, compared with treatments T8: ammonium niobate (V) oxalate + phosphate buffer and T6: ammonium oxalate, respectively. The difference observed between T7 and T8 may be related to the presence of phosphate buffer in the T8 formulation, which was absent in T7, and may have helped mitigate the adverse effects of water deficit on the root system. However, this marked reduction observed in T7 was not reflected in root length (Figure 3F) and plant height (Figure 3G) variables, since, regardless of the type of hydropriming applied to the maize seeds, the water deficit imposed on treatments T3 to T8 resulted in average reductions of 46.82% in root length and 42.25% in plant height compared with the irrigated treatments (T1 and T2).

### 2.3. Oxidative Stress and Antioxidant System Activity

**Figure 4:**
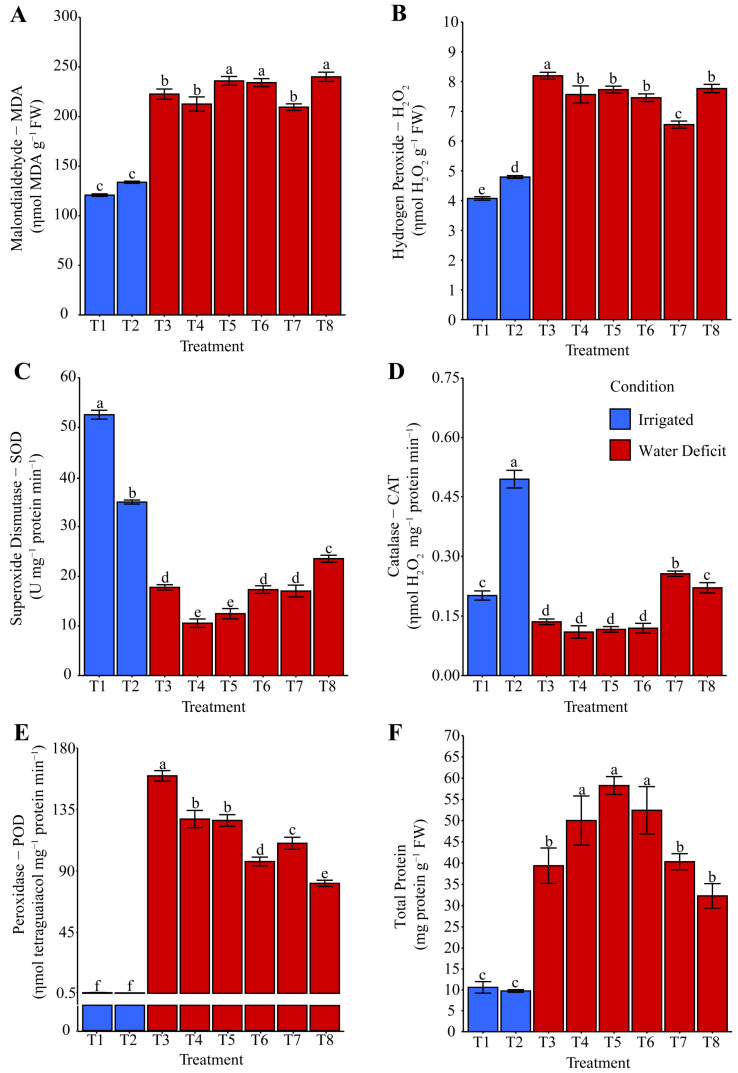
Effect of different hydropriming treatments on (**A**) malondialdehyde (MDA), (**B**) hydrogen peroxide (H_2_O_2_) contents, the activities of the antioxidant enzymes (**C**) superoxide dismutase (SOD), (**D**) catalase (CAT), (**E**) peroxidase (POD), and (**F**) total proteins in maize seedlings under water deficit. Lowercase letters indicate statistically significant differences between hydropriming treatments, as determined by the Scott–Knott test (*p* < 0.05). Bars represent the mean, and whiskers represent the associated standard error. Under irrigation conditions, treatment T1 consisted of hydropriming maize seeds with distilled water (H_2_O), while treatment T2 used a phosphate-buffer solution. Under water deficit conditions, treatments involved hydropriming with distilled water (T3), phosphate buffer (T4), ammonium oxalate (T5), a combination of ammonium oxalate and phosphate buffer (T6), ammonium niobate (V) oxalate (T7), and a combination of ammonium niobate (V) oxalate and phosphate buffer (T8).

**Figure 5:**
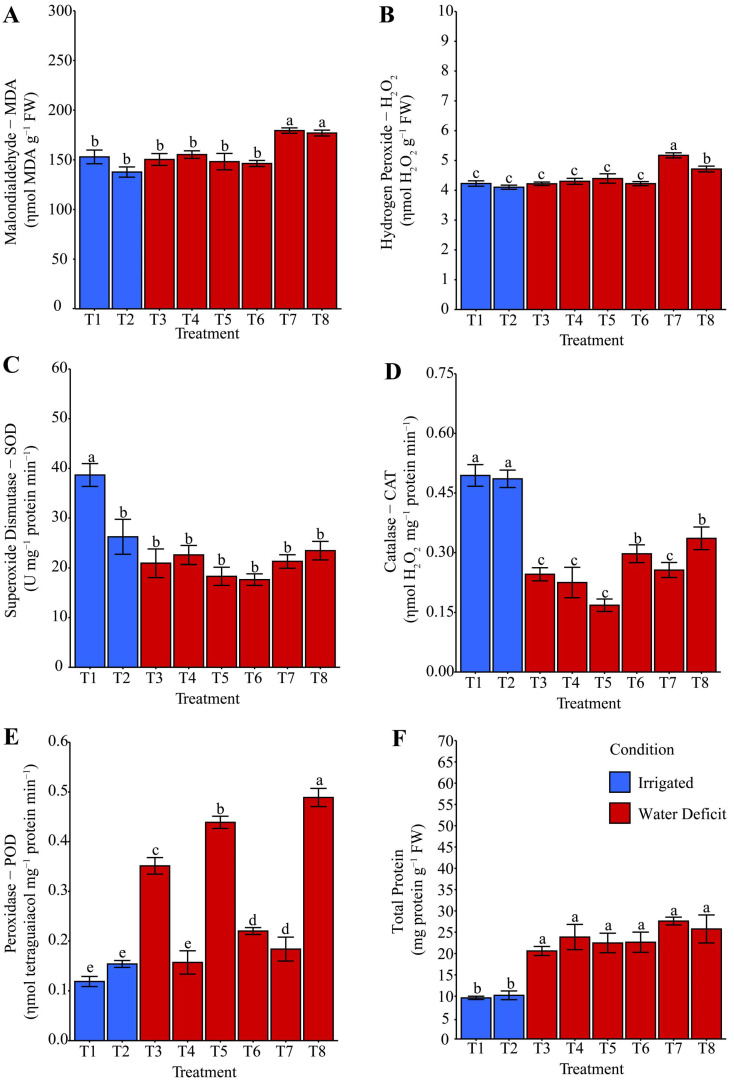
Effect of different hydropriming treatments on (**A**) malondialdehyde (MDA), (**B**) hydrogen peroxide (H_2_O_2_) contents, the activities of the antioxidant enzymes (**C**) superoxide dismutase (SOD), (**D**) catalase (CAT), (**E**) peroxidase (POD), and (**F**) total proteins in maize plants after rehydration. Lowercase letters indicate statistically significant differences between hydropriming treatments, as determined by the Scott–Knott test (*p* < 0.05). Bars represent the mean, and whiskers represent the associated standard error. Under irrigation conditions, treatment T1 consisted of hydropriming maize seeds with distilled water (H_2_O), while treatment T2 used a phosphate-buffer solution. Under water deficit conditions, treatments involved hydropriming with distilled water (T3), phosphate buffer (T4), ammonium oxalate (T5), a combination of ammonium oxalate and phosphate buffer (T6), ammonium niobate (V) oxalate (T7), and a combination of ammonium niobate (V) oxalate and phosphate buffer (T8).

The MDA content (Figure 4A), which indicates lipid peroxidation, was reduced by 40.78% in treatments T1: distilled water and T2: phosphate buffer, both under irrigated conditions, compared with treatments subjected to water deficit stress (T3: distilled water, T4: phosphate buffer, and T7: ammonium niobate (V) oxalate). The latter treatments showed, on average, 9.23% lower MDA levels than those observed in treatments T5 (ammonium oxalate), T6 (ammonium oxalate + phosphate buffer), and T8 (ammonium niobate (V) oxalate + phosphate buffer). Thus, treatments with ammonium oxalate (T5, T6, and T8) under water deficit stress promoted an average increase of 31.64% in MDA content compared with the other treatments. After rehydration, a 20.03% increase in MDA levels (Figure 5A) was explicitly observed in treatments containing niobium (T7 and T8), compared with the other treatments, including the irrigated treatments (T1 and T2).

Hydrogen peroxide (H_2_O_2_) was reduced by 15.03% in T1 (distilled water) compared with T2 (phosphate buffer), which in turn was lower (−26.89%) than in T7 (ammonium niobate (V) oxalate). Under water deficit conditions, T7 was lower (−14.09%) than T4: phosphate buffer, T5: ammonium oxalate, T6: ammonium oxalate + phosphate buffer, and T8: ammonium niobate (V) oxalate + phosphate buffer, which were on average (+7.42%) higher than T3: distilled water. Hydropriming maize seeds exclusively with distilled water (T3) showed the highest H_2_O_2_ concentration (+10.53%) compared with the other treatments under water restriction (Figure 4B). After rehydration, T7, with ammonium oxalate-niobium, was the only treatment to increase H_2_O_2_ concentration (+21.85%), followed by T8, with ammonium oxalate-niobium and phosphate buffer, which increased this variable by 11.03%, both compared with the other treatments (Figure 5B).

SOD activity (Figure 4C) increased in T1 (+50.42%) compared with T2, and T1 was higher than T8 under irrigation conditions. Under irrigation suspension, and therefore under water deficit conditions, T8, with ammonium oxalate-niobium and phosphate buffer, showed on average the highest SOD activity (+56.44%) compared with T3, T4, T5, T6, and T7. T4 matched T5 and had the lowest activity (−39.13%) for this enzyme compared with the other treatments under water deficit. After rehydration, SOD activity (Figure 5C) was only favored under irrigation in T1; T2 did not show the same pattern and instead matched the hydropriming treatments subjected to irrigation suspension, even in the presence of Nb.

CAT activity (Figure 4D) under irrigation conditions was favored exclusively by T2, whereas T1 showed the lowest activity. Under water restriction, T7 was higher (+16.01%) than T8, which, on average, was higher (+84.01%) than T3, T4, T5, and T6. After rehydration, T1 and T2 showed similar levels and stood out with the highest CAT activity, which was 92.30% higher than that of the other treatments subjected to water deficit. Treatments T6 and T8 showed, on average, 41.33% higher activity compared with the different treatments, which were also exposed to water restriction.

POD activity (Figure 4E) under water deficit was increased exclusively in T3, which was on average 25.20% higher than T4 and T5, which were 15.59% higher than T7. Moreover, T7 was 13.74% higher than T6, which in turn was 19.70% higher than T8. It is notable that under irrigation, either during the water deficit evaluation or after rehydration, T1 and T2 exhibited the lowest POD activity (Figure 4E and Figure 5E). After rehydration, T8 showed 11.38% higher POD activity compared with T5, which was 24.96% higher than T3, 73.97% higher than T6 and T7, and on average 28.65% higher than T4 (Figure 5E).

The highest protein synthesis (Figure 4F and Figure 5F) occurred under water deficit compared with irrigation conditions. Exclusively under water deficit, treatments T5, T4, and T6 were on average 43.45% higher than T3, T7, and T8.

### 2.4. Starch and Compatible Osmolytes

**Figure 6:**
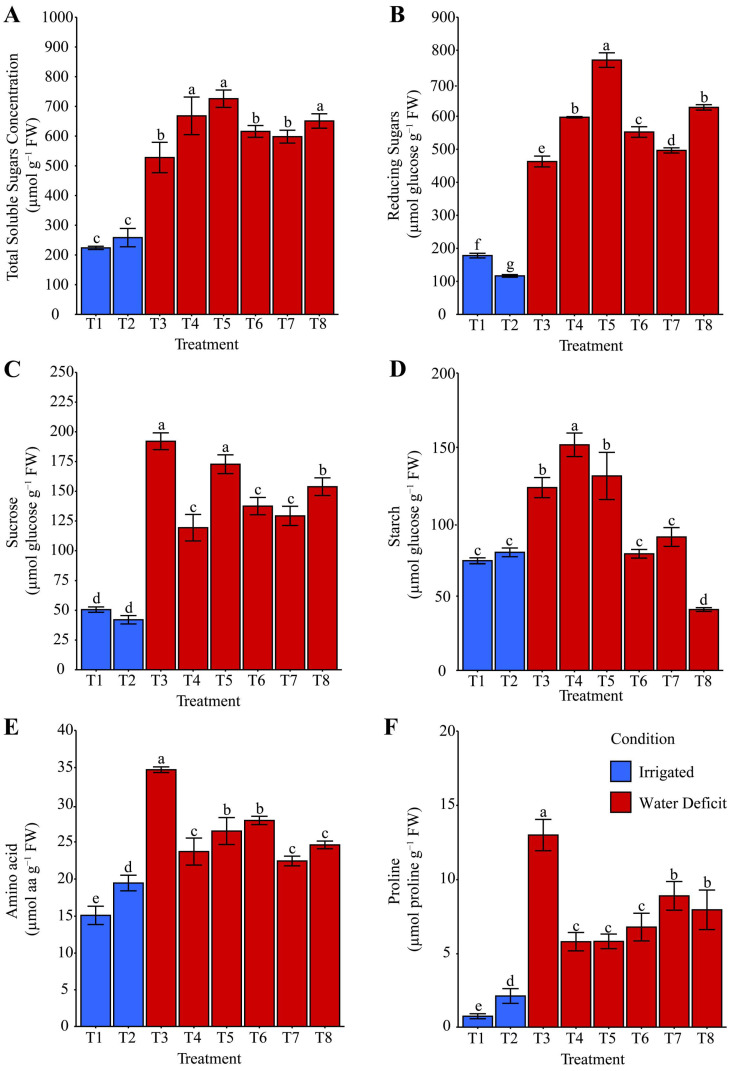
Effect of different hydropriming treatments on the levels of (**A**) total soluble sugars (TSS), (**B**) reducing sugars, (**C**) sucrose, (**D**) starch, (**E**) total free amino acids, and (**F**) proline in maize seedlings under water deficit. Lowercase letters indicate statistically significant differences among the various hydropriming treatments, as determined by the Scott–Knott test (*p* < 0.05). Bars represent the mean, and whiskers represent the associated standard error. Under irrigation conditions, treatment T1 consisted of hydropriming maize seeds with distilled water (H_2_O), while treatment T2 used a phosphate-buffer solution. Under water deficit conditions, treatments involved hydropriming with distilled water (T3), phosphate buffer (T4), ammonium oxalate (T5), a combination of ammonium oxalate and phosphate buffer (T6), ammonium niobate (V) oxalate (T7), and a combination of ammonium niobate (V) oxalate and phosphate buffer (T8).

**Figure 7:**
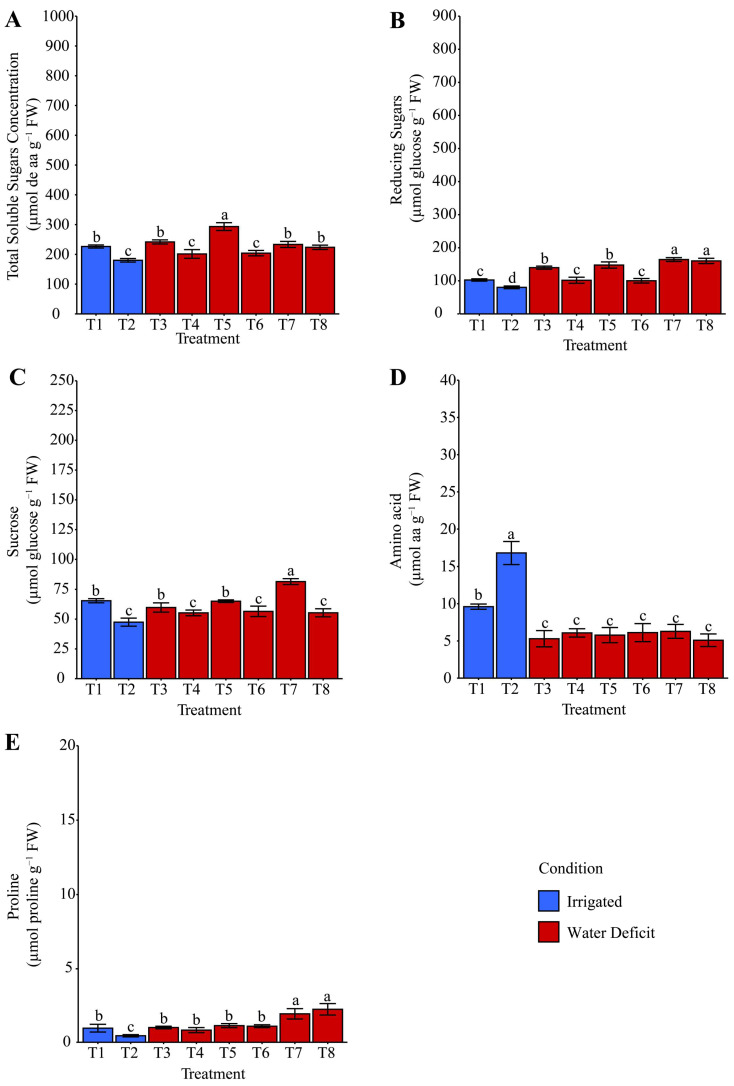
Effect of different hydropriming treatments on the levels of (**A**) total soluble sugars (TSS), (**B**) reducing sugars, (**C**) sucrose, (**D**) total free amino acids, and (**E**) proline in maize plants after rehydration. Lowercase letters indicate statistically significant differences among the various hydropriming treatments, as determined by the Scott–Knott test (*p* < 0.05). Bars represent the mean, and whiskers represent the associated standard error. Under irrigation conditions, treatment T1 consisted of hydropriming maize seeds with distilled water (H_2_O), while treatment T2 used a phosphate-buffer solution. Under water deficit conditions, treatments involved hydropriming with distilled water (T3), phosphate buffer (T4), ammonium oxalate (T5), a combination of ammonium oxalate and phosphate buffer (T6), ammonium niobate (V) oxalate (T7), and a combination of ammonium niobate (V) oxalate and phosphate buffer (T8).

Lower total soluble sugars (TSS) (Figure 6A) were observed exclusively under irrigated conditions at both 14 and 21 days of evaluation. During the period of water deficit, T8: ammonium niobate (V) oxalate + phosphate buffer, T4: phosphate buffer, and T5: ammonium oxalate were on average 17.40% higher than T3: distilled water, T6: ammonium oxalate + phosphate buffer, and T7: ammonium niobate (V) oxalate. After rehydration, TSS (Figure 7A) was 25.96% higher in T5 than in T3, T7, and T8, and these were 14.89% higher than T4 and T6. Reducing sugars (RS) (Figure 6B) were exclusively enhanced (+77.91%) by hydropriming with ammonium oxalate (T5) compared with the other treatments under water deficit, including the irrigated condition. However, this pattern was not observed after rehydration (Figure 7B), as T7 and T8 were 44.91% higher than the other treatments.

During the water deficit, sucrose (Figure 6C) was enhanced by hydropriming with distilled water and ammonium oxalate (T3 and T5) compared with T8, which contained niobium. This enhancement was higher than those of T4, T6, and T7. After rehydration, T7, with niobium and without phosphate buffer, positively contributed (+24.53%, +71.77%, and +47.36%) to the increase in sucrose content (Figure 7C) compared with T1, T2, and T8, respectively.

Starch, evaluated exclusively during water deficit (Figure 6D), showed a positive response (+19.62%) only under the hydropriming treatment with phosphate-buffer solution when compared with T3 and T5, which were higher (+50.97%) than T6 and T7. The irrigated condition (T1 and T2) showed higher (+86.77%) starch content than T8.

Amino acid concentration (Figure 6E) was generally favored (+54.04%) by hydropriming treatments, regardless of the solution used, under irrigation suspension compared with the irrigated condition. However, this pattern was reversed after rehydration (Figure 7D), as treatments subjected to water deficit had lower (−56.25%) amino acid concentration compared with T1 and T2.

Proline (Figure 6F) showed the lowest contribution, regardless of the evaluation period, under irrigated conditions (T1 and T2). Under water deficit, its concentration was favored (+54.04%) exclusively by T3 (hydropriming with distilled water) when compared with T7 and T8. However, after rehydration (Figure 7E), T7 and T8 showed higher (+108.62%) proline levels than T3.

### 2.5. Chlorophyll and Carotenoid Contents

**Figure 8:**
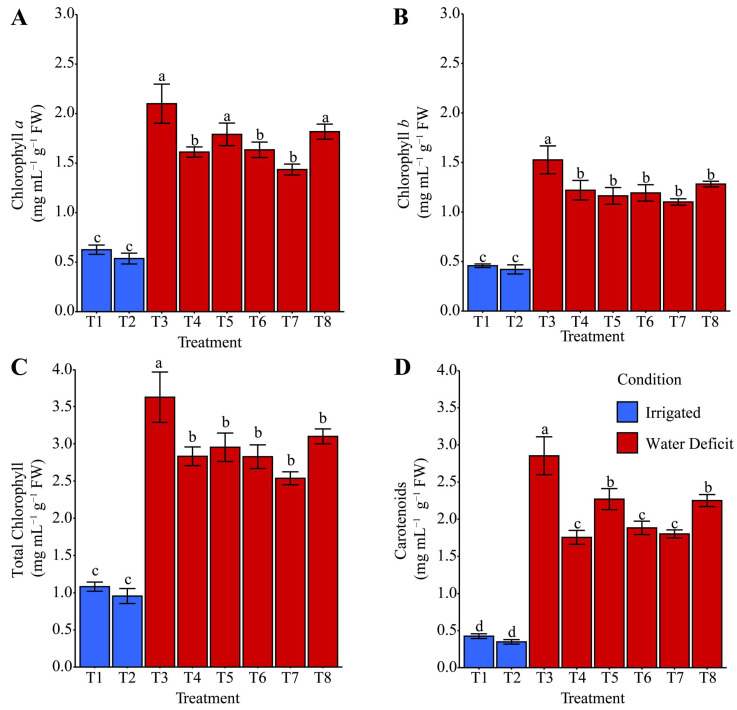
Effect of different hydropriming treatments on the contents of (**A**) chlorophyll *a*, (**B**) chlorophyll *b*, (**C**) total chlorophyll, and (**D**) carotenoids in maize seedlings under water deficit. Lowercase letters indicate statistically significant differences among the various hydropriming treatments, as determined by the Scott–Knott test (*p* < 0.05). Bars represent the mean, and whiskers represent the associated standard error. Under irrigation conditions, treatment T1 consisted of hydropriming maize seeds with distilled water (H_2_O), while treatment T2 used a phosphate-buffer solution. Under water deficit conditions, treatments involved hydropriming with distilled water (T3), phosphate buffer (T4), ammonium oxalate (T5), a combination of ammonium oxalate and phosphate buffer (T6), ammonium niobate (V) oxalate (T7), and a combination of ammonium niobate (V) oxalate and phosphate buffer (T8).

**Figure 9:**
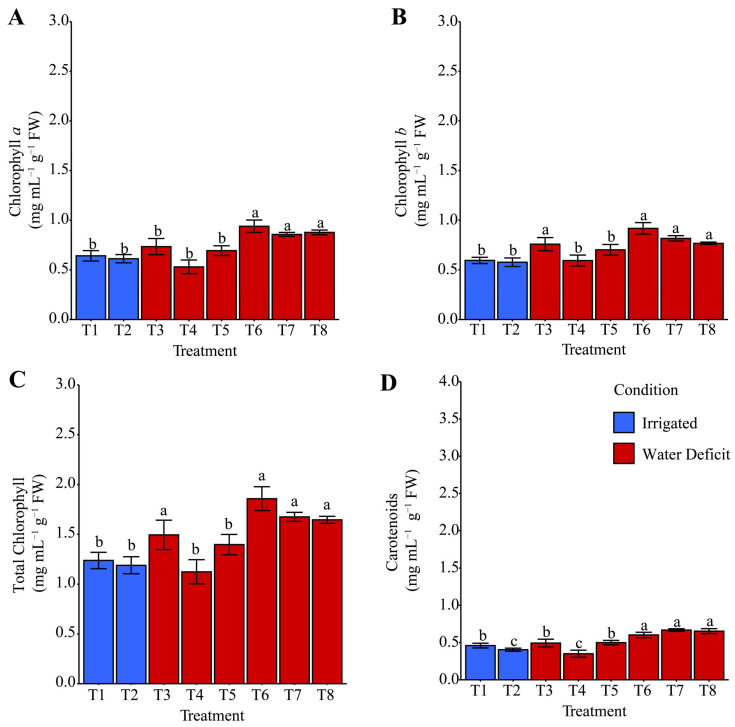
Effect of different hydropriming treatments on the contents of (**A**) chlorophyll *a*, (**B**) chlorophyll *b*, (**C**) total chlorophyll, and (**D**) carotenoids in maize plants after rehydration. Lowercase letters indicate statistically significant differences between hydropriming treatments, as determined by the Scott–Knott test (*p* < 0.05). Bars represent the mean, and whiskers represent the associated standard error. Under irrigation conditions, treatment T1 consisted of hydropriming maize seeds with distilled water (H_2_O), while treatment T2 used a phosphate-buffer solution. Under water deficit conditions, treatments involved hydropriming with distilled water (T3), phosphate buffer (T4), ammonium oxalate (T5), a combination of ammonium oxalate and phosphate buffer (T6), ammonium niobate (V) oxalate (T7), and a combination of ammonium niobate (V) oxalate and phosphate buffer (T8).

Chlorophyll *a* during the irrigation suspension (Figure 8A) was on average (+21.93%) higher for T3: distilled water, T5: ammonium oxalate, and T8: ammonium niobate (V) oxalate + phosphate buffer when compared with T4: phosphate buffer, T6: ammonium oxalate + phosphate buffer, and T7: ammonium niobate (V) oxalate, which was itself higher (168.52%) than T1 and T2. For total chlorophyll, T3 was the only treatment that increased this variable by 27.24% compared with the other treatments under water deficit conditions and by 255.54% compared with the irrigated treatments (T1 and T2). After rehydration, both chlorophyll *a* (Figure 9A) and total chlorophyll (Figure 9C) were generally increased on average (+38.73% and +33.97%, respectively) in T6, T7, and T8 compared with the other treatments; however, T3 contributed exclusively to the increase in total chlorophyll. Chlorophyll *b* and carotenoids (Figure 8B,D) showed similar behavior. The T3 treatment favored both variables compared with the other treatments under both water-deficient and irrigated conditions. After rehydration (Figure 9B,D), in addition to T3, chlorophyll *b* and carotenoids were favored by treatments T6, T7, and T8 compared with the other treatments and the irrigated condition.

### 2.6. MINI-PAM Analysis

**Figure 10:**
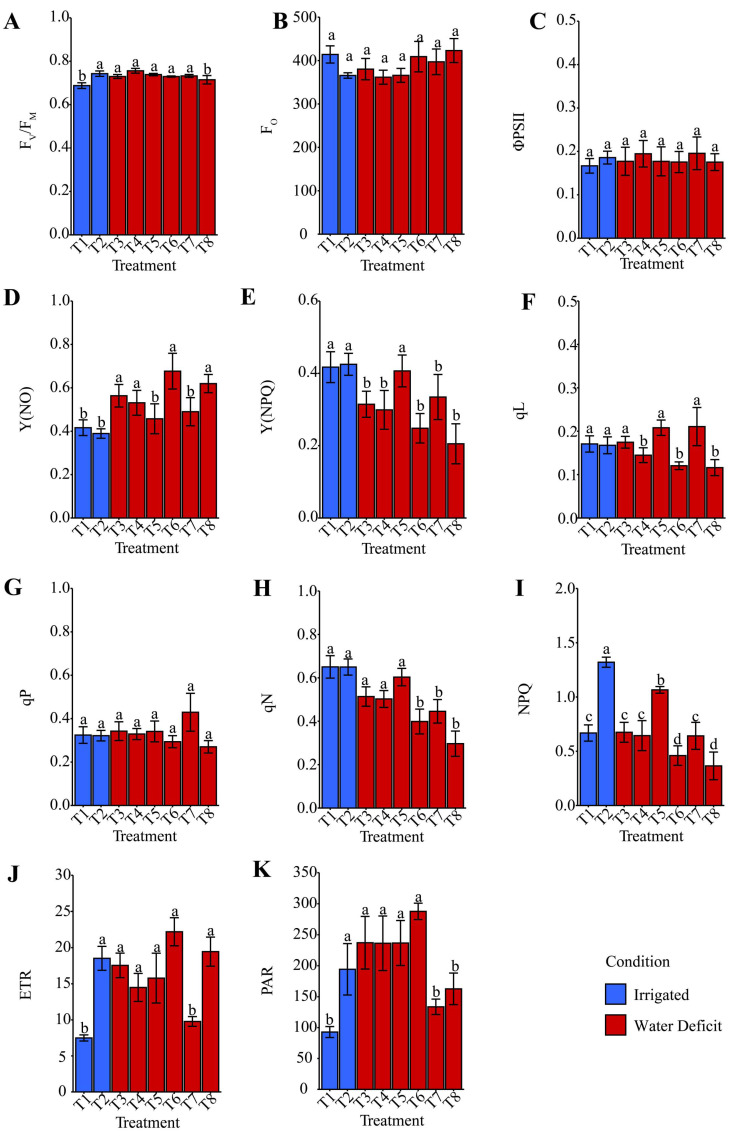
Effect of different hydropriming treatments on the quantity related to the (**A**) maximum quantum yield of PSII photochemistry (F_V_/F_M_), (**B**) minimum Chl *a* fluorescence yield in the dark-adapted state (F_O_), (**C**) effective quantum yield of PSII (ΦPSII), (**D**) quantum yield of non-regulated energy dissipation (Y(NO)), (**E**) quantum yield of regulated energy dissipation via NPQ-dependent mechanisms (Y(NPQ)), (**F**) photochemical quenching coefficient based on the fraction of open PSII reaction centers (qL), (**G**) photochemical quenching coefficient based on the ratio between open and closed centers (qP), (**H**) non-photochemical quenching coefficient (qN), (**I**) Stern–Volmer non-photochemical quenching (NPQ), and (**J**) electron transport rate (ETR) under different (**K**) photosynthetically active radiation (PAR) conditions in maize plants after rehydration. Lowercase letters indicate statistically significant differences among the various hydropriming treatments, as determined by the Scott–Knott test (*p* < 0.05). Bars represent the mean, and whiskers represent the associated standard error. Under irrigation conditions, treatment T1 consisted of hydropriming maize seeds with distilled water (H_2_O), while treatment T2 used a phosphate-buffer solution. Under water deficit conditions, treatments involved hydropriming with distilled water (T3), phosphate buffer (T4), ammonium oxalate (T5), a combination of ammonium oxalate and phosphate buffer (T6), ammonium niobate (V) oxalate (T7), and a combination of ammonium niobate (V) oxalate and phosphate buffer (T8).

The photosynthetic variables obtained using the MINI-PAM were evaluated only after the plant had rehydrated (Figure 10). These results showed that F_0_, ΦPSII, and qP (Figure 10B,C,G) did not differ statistically from each other when comparing the irrigated treatments (T1 and T2) with the treatments subjected to water deficit.

A quantity related to the maximum quantum yield of PSII photochemistry (F_V_/F_M_) (Figure 10A) was, on average, reduced by 5.00% for T1: distilled water and T8: ammonium niobate (V) oxalate + phosphate buffer, compared with the other treatments. YN0 (Figure 10D) was increased by T1 and T2: phosphate buffer, and this condition was statistically similar to treatments T5: ammonium oxalate and T7: ammonium niobate (V) oxalate + phosphate buffer, which used ammonium oxalate and ammonium oxalate–niobium, respectively, as hydropriming; this pattern was not observed for the other treatments, which reduced this indicator of photosynthetic inefficiency.

The quantum yield of regulated energy dissipation via NPQ-dependent mechanisms, Y(NPQ), as shown in Figure 10E, was favored on average (+48.51%) for the irrigated treatments (T1 and T2), which were similar to T5, the maize seeds hydroprimed with ammonium oxalate, compared with the other treatments.

The lowest photochemical quenching coefficient based on the fraction (qL), Figure 10F, was observed for T4: phosphate buffer, T6: ammonium oxalate + phosphate buffer, and T8: ammonium niobate (V) oxalate + phosphate buffer, all containing phosphate buffer, relative to the other treatments. The non-photochemical quenching coefficient (qN), as shown in Figure 10H, exhibited lower protective efficiency (−29.56%) in T6, T7, and T8 compared with the other treatments subjected to water deficit via irrigation suspension.

The results associated with Stern–Volmer non-photochemical quenching (NPQ), as shown in Figure 10I, were dynamic. Treatments T6 and T8 had the lowest values compared with T3: distilled water, T4, T7, and T1, which were lower than T5; however, T2 under continuous irrigation exhibited the highest condition (+105.71% and +97.67%) when compared with the treatments under water deficit and T1 (irrigated), respectively.

The electron transport rate (ETR), as shown in Figure 10J, indicated that T1 and T7 exhibited slow electron transport, or low photosynthesis (−52.02%), compared with the other treatments.

Additionally, the lowest photosynthetically active radiation (PAR) was observed for T1 under irrigated conditions with hydropriming in distilled water, as well as for T7 and T8, both containing Nb, averaging 45.59% lower than the other treatments (Figure 10K).

### 2.7. Multivariate Analysis

**Figure 11:**
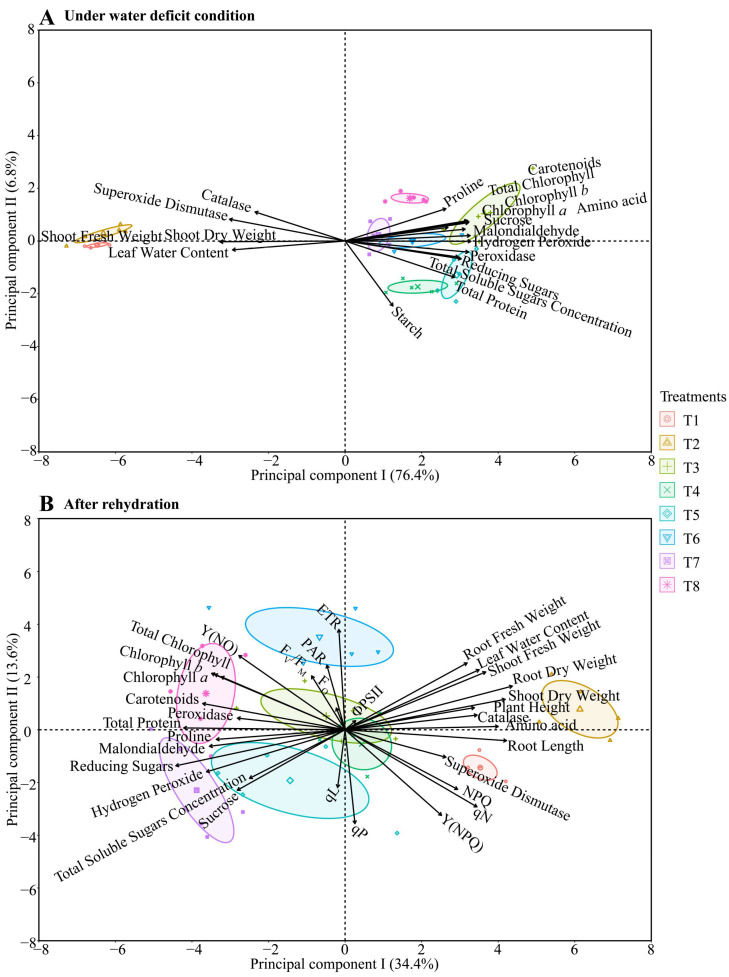
Principal component analysis related to different hydropriming treatments of maize seeds, (**A**): under water deficit conditions, and (**B**): after rehydration. ETR: Electron transport rate. PAR: Photosynthetically active radiation. F_V_/F_M_: a quantity related to the maximum quantum yield of PSII photochemistry. F_O_: Minimum Chl *a* fluorescence yield in the dark-adapted state. qL: Photochemical quenching coefficient based on the fraction of open PSII reaction centers. qP: Photochemical quenching coefficient based on the ratio between open and closed centers. Y(NPQ): Quantum yield of regulated energy dissipation via NPQ-dependent mechanisms. qN: Non-photochemical quenching coefficient. NPQ: Stern–Volmer non-photochemical quenching. Yield PSII (ΦPSII): Effective quantum yield of PSII. T1 consisted of priming maize seeds with distilled water (H_2_O), T2 with phosphate buffer. T3 involved priming with distilled water, T4 with phosphate buffer, T5 with ammonium oxalate, T6 with a combination of ammonium oxalate and phosphate buffer, T7 with ammonium niobate (V) oxalate, and T8 with a combination of ammonium niobate (V) oxalate and phosphate buffer.

In the principal component analysis (PCA) conducted on maize plants subjected to different hydropriming treatments under water deficit conditions (Figure 11A), a clear distinction was observed between the treatments maintained under irrigation (T1 and T2) and those subjected to irrigation suspension. After seven days of water deficit, a positive relationship was observed among the variables SOD, CAT, shoot fresh weight (SFW), shoot dry weight (SDW), and leaf relative water content (LWC), exclusively for the irrigated treatments.

Among the treatments subjected to water deficit (Figure 11A), T3 (distilled water) and T8 (ammonium niobate (V) oxalate + phosphate buffer) exhibited distinct behaviors. Treatment T3 promoted higher levels of chlorophyll *a*, *b*, and total chlorophyll, as well as sucrose, total amino acids, MDA, and H_2_O_2_, suggesting a physiological stress response. In contrast, T8 was associated with an increased proline concentration, a key osmoprotectant solute.

In the PCA conducted after the rehydration period (Figure 11B), a more precise distinction among treatments was observed. The irrigated treatments remained segregated from the others, with T1 (distilled water) associated with higher SOD activity and T2 showing a positive relationship with root dry weight (RDW), shoot dry weight (SDW), plant height (PH), CAT activity, and amino acid content. Under water deficit conditions, treatments T3 (distilled water) and T4 (phosphate buffer) exhibited similar behavior. At the same time, T5: ammonium oxalate showed a positive relationship with total soluble sugars and sucrose, in addition to being associated with the qL parameter (Photochemical quenching coefficient based on the fraction of open PSII reaction centers).

Also in Figure 11B, treatment T6, ammonium oxalate + phosphate buffer, showed a positive relationship between the electron transport rate (ETR) and photosynthetically active radiation (PAR). The treatments T7: ammonium niobate (V) oxalate and T8, which involved the application of niobium (Nb) during seed priming, displayed specific responses after severe stress followed by rehydration. T7 (ammonium oxalate-niobium) showed a positive relationship between reducing sugars and H_2_O_2_. In contrast, T8 (a combination of ammonium oxalate, niobium, and phosphate buffer) exhibited a positive relationship among the quantum yield of non-regulated energy dissipation (Y(NO), chlorophylls *a*, *b*, and total, carotenoids, POD activity, and total protein content.

## 3. Discussion

The germination speed index (GSI) is a key variable for assessing seed vigor, as it combines information on both the rate and the time required for germination to occur. In the present study, GSI (Figure 1) was evaluated to determine whether hydropriming treatments influenced the emergence rate of maize seedlings. Overall, it is evident that the priming technique can induce strategic mechanisms in seeds, promoting early metabolic activation and preparing the antioxidant and osmotic systems to respond more efficiently to water stress. Singh et al. [35] emphasized in their review of seed treatment techniques in field crops that germination and seedling emergence represent critical stages in the developmental cycle. The authors emphasized the importance of adopting strategies such as priming, particularly for their effectiveness in regions with adverse environmental conditions, including high temperatures and drought.

Still referring to Figure 1, the highest GSI values were observed for T5, T7, and T8, all of which involved solutions containing ammonium oxalate with or without niobium (Nb). T5 showed a significant increase in germination speed, suggesting that nitrogen supply in the ammonium form may favor the activation of germinative metabolism. Additionally, T7 and T8, which included ammonium niobate (V) oxalate, showed a higher GSI, suggesting a possible biostimulant effect of Nb. This effect may be related to the element’s ability to modulate metabolic and antioxidant processes during germination, thereby promoting greater initial seedling vigor. Seed germination is characterized as a complex process that begins when the seed absorbs water after the drying and maturation period, reactivating metabolism and preparing the cells for seedling growth. During germination, several physiological and biochemical reactions occur, including the activation of carbohydrate metabolism, with starch serving as the primary source of energy. Additionally, hormones involved in development and oxidative cell control also play crucial roles [36].

Although still scarce in the literature, some evidence indicates that transition metals, when applied at low concentrations, can modulate physiological and enzymatic processes in plants, including during germination [34]. As Nb belongs to the class of transition metals, specifically to group 5 of the periodic table according to the IUPAC classification [37], and is classified as a trace element [17], the results obtained in this study contrast with those reported by Chandrasekar et al. [38], who indirectly evaluated the toxicity of dyes degraded by composites containing tantalum oxide (Ta_2_O_5_) on the germination speed index (GSI) of Vigna radiata (mung bean) seeds. Since Nb and Ta are elements often found together in nature due to their similar properties and atomic radii [39], it is noteworthy that those authors did not observe any toxic effect of the Ta_2_O_5_-containing composite on seed germination. Germination was considered reasonable and comparable to the water control, suggesting that the presence of tantalum was associated with low or no residual toxicity.

Additionally, in another study, Alcântara et al. [40] reported an increasing trend in GSI of maize seeds treated with ascorbic acid. These authors found that increasing the antioxidant concentration promoted a higher germination percentage and speed, as well as a reduction in the time required for emergence, compared with the control. Thus, the GSI results from the present study (Figure 1) suggest that Nb may play a role in inducing antioxidant mechanisms, thereby conferring greater cellular stability. The exclusion of phosphate-buffer solution effects supports this. The fact that T7 and T8 presented equal and higher contributions to GSI indicates that the phosphate-buffer solution did not provide additional benefits. This condition is reinforced by the observation that treatments T2, T4, and T6, which also included phosphate-buffer solution but without Nb, exhibited lower GSI.

The physiological and morphological parameters evaluated under the adverse effects of water deficit showed significant reductions in shoot fresh weight (FW), shoot dry weight (DW), and leaf water content (LWC), as shown in Figure 2A, Figure 2B and Figure 2C, respectively. Regardless of the hydropriming solution, there was a substantial reduction in shoot biomass. This fact is associated with reduced cell turgor and lower leaf expansion capacity under limited water availability [41]. Leaf water content (LWC) is a key indicator of a plant’s water status and physiological integrity under adverse conditions, such as drought [42]. The reduction in LWC suggests a limitation in water absorption and transport, which negatively compromises metabolic and photosynthetic processes. After rehydration, the minor losses observed in T1, T3, and T6 for FW, DW, and LWC (Figure 3A, Figure 3B and Figure 3C, respectively) compared with T2 indicate a possible mitigation of stress effects due to the specific priming-associated responses. Treatment T6, which involved seed imbibition in ammonium oxalate with phosphate buffer, performed better than T7: ammonium niobate (V) oxalate alone. This condition suggests that the presence of phosphate buffer may have contributed to better ionic balance or helped maintain the biochemical stability of membranes under stress.

However, it is possible that the phosphate buffer neutralized part of the osmoprotective or biostimulant effect of Nb through chemical complexation interactions, as it was used to adjust the pH of the priming solution. This pH adjustment may have altered the chemical form or activity of Nb. Supporting this, Ferreira et al. [43] argue in their review that the adequacy of pH buffers in biological, biochemical, and environmental studies, as well as their interaction with metal ions, necessitates a choice of buffer that extends beyond selecting the correct pH range. Known interactions between buffers, such as metal–buffer complexation and biological effects, must also be considered, as they may limit the bioavailability of elements. Nevertheless, our results also suggest a lack of protective effect against drought when Nb was combined with phosphate buffer, or that the presence of Nb may have generated additional oxidative stress, compromising the seedlings’ water balance.

Biomass is a key indicator of plant growth and is widely used and validated to assess plant development [44,45]. However, under water deficit conditions, it is common to observe reduced maize growth due to decreases in leaf area and biomass [41,46]. Evaluating these variables during water deficit, as well as after rehydration, enables an understanding of both the impacts of stress and the plants’ recovery capacity in response to physiological conditioning strategies.

The contrasting effects of Nb priming, which increased GSI in T5, T7, and T8 (Figure 1) but reduced biomass under water deficit in T7 and T8 (Figure 2 and Figure 3), reflect distinct physiological responses across developmental phases. During germination, Nb likely enhances initial metabolic activation and antioxidant defenses [34]. However, under water deficit, Nb may induce ionic toxicity or excessive reactive oxygen species (ROS), impairing cell expansion and biomass accumulation [41,47]. Trace metals at low doses often promote germination but cause toxicity during growth [48]. The acidic pH of T7 (3.571) and high electrical conductivity (EC) of T8 (70,641.1 µS cm^−1^) (Table 1) likely exacerbated these adverse effects, as discussed below.

The effects of Nb seed priming on maize germination, biomass, and oxidative stress may have been influenced by variations in pH and EC of the priming solutions containing ammonium niobate (V) oxalate (C_4_H_4_NNbO_9_) and, in some treatments (T2, T4, T6, T8), phosphate buffer. The acidic pH of T7 (3.571), Table 1, compared to the near-neutral pH of T2, T4, T6, and T8 (6.247–7.046) may have affected seed hydration, enzymatic activity, or membrane permeability, contributing to increased GSI in T5, T7, and T8 (Figure 1) or elevated oxidative stress under water deficit (Figure 4 and Figure 5). High EC in T8 (70,641.1 µS cm^−1^) and T5/T6 (16,004.9–18,079.9 µS cm^−1^), versus T7 (1860.9 µS cm^−1^) and T1/T3 (72.0 µS cm^−1^) (Table 1), likely altered osmotic balance, reducing biomass in T7 and T8 (Figure 2 and Figure 3). Phosphate buffer-Nb complexation may have reduced Nb bioavailability [43], thereby limiting the benefits of GSI in T2, T4, and T6. Although measured before priming, pH and EC were not controlled, a limitation suggesting that T7’s acidic pH and T8’s high EC exacerbated toxic and osmotic effects. Future studies should control for these variables [49] to isolate the impact of Nb accurately.

**Figure 12:**
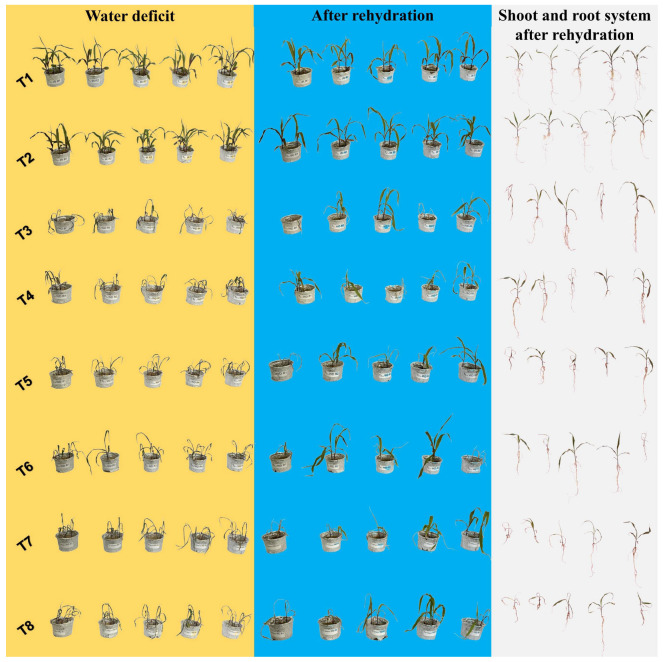
Visualization of the effects of different hydropriming treatments on maize seeds under water deficit conditions and after rehydration, including aspects of the shoot and root system. T1 consisted of maize seed priming with distilled water (H_2_O), T2 with phosphate buffer. T3 involved priming with distilled water, T4 with phosphate buffer, T5 with ammonium oxalate, T6 with a combination of ammonium oxalate and phosphate buffer, T7 with ammonium niobate (V) oxalate, and T8 with a combination of ammonium niobate (V) oxalate and phosphate buffer.

Figure 12 presents a photographic sequence of the treatments throughout different phases of the experiment: water deficit (yellow column), rehydration (blue column), and evaluation of the shoot and root system after rehydration (white column). An apparent visual variation in development between treatments can be observed, with T1 and T2 standing out due to their better vigor and leaf coloration, as they were irrigated throughout the experiment. During the water deficit period, wilting and leaf senescence symptoms were evident, especially in treatments T3, T7, and T8. After rehydration, visible recovery was noted, with resumed growth and intensified leaf coloration, particularly in T5 and T6. These results are consistent with principal component analysis (PCA), which revealed a clear separation between treatments based on water conditions and the hydropriming solutions used, with 70% of the total data variance significantly explained by the two principal components (Figure 11).

Root biomass (Figure 3D,E) showed that maize seed hydropriming with isolated ammonium niobate (V) oxalate (T7) significantly reduced RFW and RDW compared with T8 and T6. This result contrasts with the positive effects of Nb on GSI (Figure 1). Additionally, it suggests that the isolated presence of Nb, without a phosphate buffer, was insufficient to promote root system development under water deficit conditions, possibly due to ionic toxicity or the absence of stabilizing components in the priming solution. Our results suggest that Nb influenced physiological processes when applied as seed priming. The study by da Silva and de Oliveira [50] shows that the application of commercial products containing micronutrients for seed treatment in maize hybrid RB9110 affects early plant growth, depending on the concentration and chemical form used. Thus, the presence of Nb, especially after rehydration (Figure 3), interfered with enzymatic activities and reserve mobilization during germination, compromising biomass accumulation without necessarily altering plant height (Figure 3G) and root length (Figure 3F). It is suggested that morphogenetic processes related to cell elongation were less affected or only partially recovered after rehydration.

Maia Júnior et al. [51] observed similar behavior in sugarcane plants treated with glycine betaine, where leaf and root dry mass showed smaller reductions in treated plants compared with those in the untreated ones. Stem mass, however, was reduced regardless of treatment, indicating a lower protective effect of the compound in this plant region. Root dry mass followed a pattern similar to that of leaves, with an 18% reduction in stressed plants without treatment, whereas those treated with one or two applications showed much smaller reductions, around 5%. The accumulated losses in different plant parts were reflected in the total dry mass, resulting in a 15.1% reduction in plants without glycine betaine. In contrast, reductions of 5.8% and 5.9% were observed in treatments with one and two applications, respectively. Moreover, the root/shoot ratio and leaf mass ratio were not significantly altered by either water deficit or treatments, indicating that relative biomass allocation was preserved.

In disagreement with our results, the study by Moterle et al. [52] evaluated three maize cultivars under water stress induced by KCl, highlighting the superiority of cultivar BRS-Angela compared with cultivars IAC 112 and Zélia. Regarding primary root length, no significant differences were observed between cultivars BRS-Angela and IAC 112 under control (0 MPa), −0.1 MPa, and −0.9 MPa treatments. However, at −0.3 MPa, cultivar BRS-Angela exhibited greater radicle length, indicating a higher root development capacity under moderate water stress. For shoot length, cultivar IAC 112 showed the highest values under control, −0.1 MPa, and −0.3 MPa treatments. On the other hand, under more negative osmotic potentials (−0.6 and −0.9 MPa), no significant differences were observed among cultivars.

The analysis of oxidative stress under both water deficit (Figure 4) and after rehydration (Figure 5) complements the biomass observations. Malondialdehyde (MDA), a marker of lipid peroxidation and therefore oxidative stress, revealed that hydropriming treatments T5, T6, and T8 were not efficient in mitigating the effects of oxidative damage (Figure 4A). Furthermore, after rehydration, Nb-containing treatments (T7 and T8) indicated that this element is associated with intensification of oxidative processes during water recovery, as they contributed to increased MDA concentration (Figure 5A). Hydrogen peroxide (H_2_O_2_), a ROS acting as a cellular signal under adverse conditions, was higher in T3 compared with other treatments under water deficit (Figure 4B). This condition suggests that in the absence of bioactive ions, the activation of the antioxidant system was inefficient. After irrigation, Nb-based hydropriming solutions were the main contributors to the increase in this signaling molecule. This result suggests that Nb may alter the redox balance and act as a cellular pro-oxidant.

The H_2_O_2_ results in relation to Nb explain why T8 showed greater superoxide dismutase (SOD) activity under stress conditions (Figure 4C). This result suggests a potential effect of Nb on the activation of this enzyme, which is considered the first barrier of defense in the antioxidant system. The increase in SOD reflects the need to eliminate the superoxide anion, which is generated in high amounts under adverse conditions, and its conversion into H_2_O_2_. Consequently, T7 and T8 exhibited higher catalase (CAT) activity (Figure 4D), suggesting a direct response to counteract H_2_O_2_ accumulation promoted by Nb, especially after rehydration (Figure 5B). Our oxidative stress results are consistent with Figure 11, as PCA clearly shows that MDA, H_2_O_2_, and POD have a strong relationship with T3, T6, and T7. In contrast, antioxidant protection and adaptation to water and osmotic stress were more associated with T8.

Superoxide dismutase activity represents the first barrier of antioxidant defense, converting O_2_^−^ into H_2_O_2_. Increased CAT indicates efficient elimination of H_2_O_2_, which is particularly significant under conditions of high ROS production. H_2_O_2_ acts both as a stressor and as a signaling molecule regulating many physiological mechanisms essential for growth and development under both normal and stress conditions [31,53]. Hendges et al. [47] reported a significant increase in MDA in maize under drought, as well as up to a sixfold increase in CAT activity, reinforcing its antioxidant role. In our study, however, Nb-containing treatments (T7 and T8) exhibited distinct responses, characterized by higher MDA and proline levels after rehydration (Figure 5A and Figure 7E) and differential modulation of CAT and SOD (Figure 4D and Figure 5C), indicating Nb-specific effects beyond the general drought response. Xu et al. [54] conducted a study with *Poa pratensis* under water deficit, evaluating SOD and CAT activities. Peroxidase (POD), however, showed the opposite trend to SOD. The fact that T7 and T8 reduced POD relative to T3 (Figure 4E) suggests that Nb-primed seeds did not need to reinforce structural defenses against oxidative damage, since this enzyme was not activated.

The mechanisms by which Nb affects maize physiology remain poorly understood due to the limited literature available. The increase in the GSI observed in T5, T7, and T8 (Figure 1), together with the elevated oxidative stress in T7 and T8 (Figure 4 and Figure 5), suggests that Nb may modulate ROS signaling by stimulating the activity of antioxidant enzymes (SOD, CAT) during germination, while inducing excessive ROS production under water deficit conditions [47]. In addition, Nb may interfere with mineral uptake, indirectly affecting photosynthesis, as evidenced by the photoinhibition observed in T8 (Figure 8, Figure 9 and Figure 10) [55]. It is also suggested that Nb interacts with the abscisic acid (ABA) signaling pathway, thereby enhancing responses to water stress [56]. These mechanisms may have been further intensified by the acidic pH in T7 (3.571) and the high electrical conductivity in T8 (Table 1). Nonetheless, such implications still require further investigation.

Additionally, the PCA (Figure 11) shows that even under water deficit and after rehydration, the combination of phosphate buffer and Nb (T8) contributed to a balanced antioxidant response, enhancing defense mechanisms. This condition explains why T8 was positioned differently from the other treatments, associated with variables reflecting a more efficient stress response. The higher protein synthesis under water deficit in T5, T4, and T6 (Figure 4F) indicates the induction of defense proteins. This scenario shows that ammonium oxalate, with or without phosphate buffer, promoted an efficient adaptive response.

Osmoprotective accumulation was evident in T8, T4, and T5 due to increased total soluble sugars (TSS) during stress (Figure 6A). However, after rehydration, only T5 remained high, suggesting the maintenance of energy reserves, as T5 not only accumulated TSS but also preserved it post-stress (Figure 7A), which may have favored plant recovery, as shown in Figure 12. Nb without phosphate buffer (T7) was the only treatment that increased sucrose levels after rehydration (Figure 7C). This result suggests that Nb alone promoted a faster and more efficient metabolic recovery after stress.

Redillas et al. [57] reported a positive correlation between soluble sugar accumulation and higher plant tolerance to osmotic stress. Similarly, Chaves Filho and Stacciarini-Seraphin [58] observed an increase in sucrose in the leaves and roots of forage species under water deficit, mainly due to the accumulation of reducing sugars. Timpa et al. [59], working with cotton under six levels of osmotic stress, reported sucrose accumulation under severe stress (more negative osmotic potentials), likely linked to enhanced osmolyte translocation to leaves for osmotic adjustment.

Starch, as a reserve source, was favored by phosphate buffer hydropriming during stress (Figure 6D), suggesting energy stabilization. In contrast, continuously irrigated treatments showed greater degradation of this compound. Free amino acids decreased in all treatments after rehydration (Figure 7D), indicating their use in metabolic recovery. Proline was enhanced during stress by T3 (Figure 6F), but after rehydration, T7 and T8 (with Nb, regardless of phosphate buffer presence) showed greater accumulation. Additionally, PCA (Figure 11) highlights T8’s contribution to osmotic adjustment through stronger relationships between proline and TSS. These results suggest that Nb effectively promotes the accumulation of an important osmoprotectant even after stress.

Regarding pigments (chlorophyll *a*, *b*, total, and carotenoids), T3 was the most effective treatment in maintaining and restoring photosynthetic integrity under stress (Figure 8). However, after rehydration, Nb treatments showed higher chlorophyll *a* and carotenoids compared with T3, while chlorophyll *b* and total chlorophyll behaved similarly, potentially reflecting enhanced photosynthetic capacity. Peloso et al. [60] reported reductions in chlorophyll (*a* + *b*) of approximately 16% and 10% under 30% and 60% water availability, respectively, compared with well-watered plants (100%). They also observed a significant decrease in the chlorophyll *a*/*b* ratio with reduced water, whereas carotenoid content remained stable despite stress intensity.

Photosynthetic variables after rehydration showed different adaptive strategies among hydroprimings. The statistical similarity between stressed hydroprimings and irrigated treatments for Minimum Chl *a* fluorescence (F_O_), Effective quantum yield of PSII (ΦPSII), and photochemical quenching coefficient based on the ratio between open and closed centers (qP) (Figure 10B,C,G) suggests partial or complete recovery of photosynthetic activity. This result aligns with pigment stability results (Figure 9). The reduction in maximum PSII quantum efficiency (F_V_/F_M_), particularly in T8 (Figure 10A), suggests a slight residual photoinhibition even after rehydration. However, this did not significantly compromise photosynthetic performance, considering the pigment-related gains (Figure 8 and Figure 9), indicating greater resilience following stress. T1, T2, T5, and T7 exhibited a lower quantum yield of non-regulated energy dissipation (Y(NO)), which is associated with photosynthetic inefficiency (Figure 10D). This result supports earlier findings for phosphate buffer and phosphate buffer + Nb treatments, which displayed better photochemical regulation.

Peloso et al. [60] observed a 63% reduction in ΦPSII under severe water deficit (30% water availability) compared with well-watered plants, indicating reduced light-use efficiency for photosynthesis. This condition, combined with lower chlorophyll concentrations, indicated impaired light absorption and energy utilization, thereby limiting electron transport in PSII [61].

The higher quantum yield of regulated energy dissipation via NPQ-dependent mechanisms (Y(NPQ)) in irrigated treatments and in stressed T5 (Figure 10E) reflects greater photoprotective capacity. This result suggests ammonium oxalate preserved photoprotection even under stress. The photochemical quenching coefficient based on the fraction of open PSII reaction centers (qL) was reduced by phosphate buffer treatments (T4, T6, T8) (Figure 10F), suggesting interference in photochemical recovery. The non-photochemical quenching coefficient (qN) decreased in Nb treatments (T7 and T8) (Figure 10H), indicating weaker defenses against excess absorbed light. However, this did not align with the enzyme activity results from Figure 4 and Figure 5. Stern–Volmer non-photochemical quenching (NPQ) was higher in T3 and T5 (Figure 10I), indicating a more active non-photochemical protection mechanism. This result suggests that although ΦPSII recovered, greater dissipation of excess energy was required to avoid PSII oxidative damage, consistent with Figure 4, Figure 5, Figure 6 and Figure 7. The electron transport rate (ETR) was enhanced in T6, T7, and T8, indicating efficient electron flow even after stress (Figure 10J), which supports the integrity of photosynthesis. Reduced photosynthetically active radiation (PAR) in T7 and T8 (Figure 10K) indicates light absorption limitations, suggesting phototoxic effects or inhibition of photosynthetic components.

The suggested phototoxic effects are supported by the significant reduction in PAR (Figure 10K), accompanied by increased MDA (Figure 5A) and H_2_O_2_ (Figure 5B) in T7, indicating oxidative damage to photosynthetic structures and a reduction in root biomass (RFW and RDW, Figure 3D,E), characterizing evident physiological stress. In contrast, T8 also showed a decrease in PAR (Figure 10K). However, the presence of phosphate buffer partially mitigated the stress, preserving chlorophylls (Figure 9A,C), total protein (Figure 5F), and promoting osmoprotective responses such as proline (Figure 7E) and POD activity (Figure 5E). Thus, hydropriming exclusively with Nb can induce phototoxic effects, whereas the addition of phosphate buffer contributes to modulating these effects.

Peloso et al. [60] also reported a significant increase in non-photochemical dissipation mechanisms. Increases of 9% and 10% in Y(NPQ) under 30% water availability compared with 60% and 100% indicate that a greater portion of excitation energy was dissipated as heat. This condition means that a severe water deficit favors non-photochemical over photochemical dissipation, negatively impacting photosynthesis. Moreover, the 15% increase in Y(NO) demonstrated that water deficit effectively increased non-regulated energy dissipation, representing constitutive energy losses in PSII antennae, as well as via fluorescence, further enhancing non-photochemical pathways.

Niobium exhibits relatively high mobility under humid environmental conditions. In edible plants, the typical range is 0.02 to 1.1 mg kg^−1^ [17]. The exact role of Nb in plant metabolism has not yet been fully elucidated; however, previous studies suggest that it may play a significant role in plant development. Ray et al. [25] observed Nb bioaccumulation in mangroves during growth, indicating a connection between atmospheric CO_2_ sequestration and Nb accumulation. The same authors pointed out that increasing levels of atmospheric CO_2_ may lead to greater Nb accumulation in plants. This scenario highlights the importance of assessing the effects of Nb on plant metabolism, considering its uptake and accumulation. Currently, the literature indicates that the mechanisms of Nb action in plants have not been fully elucidated, particularly for major crop species. Thus, comprehensive studies on the potential of this element as a plant growth biostimulant, as well as its toxicity, are urgently needed.

Therefore, future research should prioritize the sustainable management of Nb accumulation, aiming to better understand the processes that regulate the availability and mobility of this trace element in plants, thereby predicting its transport and elucidating its biogeochemical cycle. It is also essential to determine the optimal application rate, evaluate different application methods such as foliar spraying, and investigate Nb interactions with other nutrients that may influence its bioavailability, as well as its environmental impacts. Such studies are crucial to prevent potential deleterious effects on human health and environmental quality [62].

## 4. Materials and Methods

### 4.1. Experimental Condition

The experiment was conducted in a greenhouse at the Department of Soil Science (21°13′33.2″ S, 44°58′43.3″ W) of the Federal University of Lavras (UFLA), located in Lavras, Minas Gerais, Brazil. Environmental conditions inside the greenhouse were controlled, maintaining a daytime temperature of 28 °C ± 2 °C and a nighttime temperature of 15 °C ± 2 °C, with a 12 h photoperiod and a relative humidity of 60–70%. Hybrid maize seeds Itapuã 700 (CD 308) were used. Seeds were subjected to pre-germination with various solutions to assess the effect of hydropriming. The amount of solution and seeds was based on a ratio of 60 seeds per 200 mL of prepared solution. Each solution was kept in beakers covered with aluminum foil for 24 h to prevent external interference. After this period, the soaked seeds were separately dried at room temperature for 1 h on paper towels, achieving the optimal moisture for sowing, as described by Nciizah et al. [63].

After hydropriming, eight maize seeds from each solution were sown at a depth of 5 mm in 500 g of previously sieved, washed, and oven-dried sand (at 100 °C). Irrigation with distilled water was determined according to the seed analysis guidelines [64], using 500 g of dry sand as a reference. The water volume was adjusted according to the species, set at 60% of the water-holding capacity for grasses, such as maize, corresponding to 120 mL per 500 g of medium-textured sand. The sand was placed in transparent plastic pots wrapped in aluminum foil to maintain humidity and ensure stable experimental conditions. Maize plants were grown for 21 days, with the Germination Speed Index (GSI) monitored daily until treatments reached a seedling emergence rate of ≥50%. This criterion ensured uniform early plant development and minimized growth variability between treatments, in accordance with OECD protocol 208/2006.

### 4.2. Experimental Design and Treatments

The experiment was conducted using a randomized block design with five replications and eight treatments, four of which served as controls. Two positive controls (T1: distilled water; T2: phosphate buffer) were maintained under irrigation throughout the experiment, while two negative controls (T3: distilled water; T4: phosphate buffer) were subjected to water deficit followed by rehydration (Table 1). The remaining treatments (T5 to T8), also exposed to water deficit followed by rehydration, corresponded to different hydropriming solutions, as described in Table 1, aiming to isolate the effect of niobium (Nb), the focus of this study.

Considering that the commercial reagent used was ammonium niobate (V) oxalate (C_4_H_4_NNbO_9_, Sigma-Aldrich^®^ St. Louis, MI, USA), treatments were included to isolate the effects of ammonium oxalate and the phosphate buffer (K_2_HPO_4_), which was used to stabilize the solution pH. The need for a phosphate buffer was evident in preliminary readings, where the solution containing only C_4_H_4_NNbO_9_ had a pH of 3.571 and an electrical conductivity of 1860.9 µS cm^−1^. In contrast, the addition of the buffer raised the pH to 6.247 and the conductivity to 70,641.1 µS cm^−1^. Therefore, a secondary objective of the experiment was to isolate the possible effect of pH changes caused exclusively by the presence of Nb.

To evaluate maize performance under water deficit, some analyses were performed both during the stress period (irrigation suspension) and after rehydration. Each experimental block consisted of eight pots with five replications, totaling 40 experimental units.

During the germination period and up to the seventh day after sowing, all treatments were irrigated as needed, with the pots weighed every 24 h. Seedling emergence was monitored from the first day after sowing, constantly at the same time of day, and continued until emergence reached a stable level, defined as no significant increase in the number of emerged seedlings for at least two to three consecutive days, a period lasting seven days. Subsequently, the Germination Speed Index (GSI) was calculated according to the formula described by Ávila et al. [65] and Maguire [66]:

(1) $$\mathrm{GSI}\;=\;\frac{N1}{D1}+\;\frac{N2}{D2}+\;\frac{N3}{D3}+\ldots \;\frac{Nn}{Dn},$$

where *N* represents the number of seedlings emerged on the day of counting, and *D* is the number of days after sowing on which the count was made.

### 4.3. MINI-PAM Analysis and Leaf Sample Collection

On the seventh day after sowing, the plants were thinned, leaving two specimens per pot. On the eighth day, water deficit was induced by suspending irrigation, which was applied only to treatments T3 through T8. This water deficit was fully established by the 14th day after sowing, seven days after irrigation suspension, and confirmed by monitoring the maximum quantum yield of photosystem II (PSII) using a MINI-PAM modulated light fluorometer (Walz, Effeltrich, Germany), where a considerable reduction in F_V_/F_M_ values (<0.50) indicated stress.

After inducing water deficit, one plant per pot was collected, with only the aerial part removed. Roots were not harvested at this stage to avoid damaging the remaining plants, which likely had intertwined root systems. After the stress period, plants were rehydrated, and seven days later (21 days after sowing), a significant increase in F_V_/F_M_ values (>0.60) was observed. At this time, the remaining plants were collected in their entirety, including both the aerial parts and root systems.

Under water deficit (14 days after sowing), the third fully expanded leaf was collected. After rehydration (21 days after sowing), the fifth fully expanded leaf was harvested. These leaves were immediately immersed in liquid nitrogen and stored at −80 °C in an ultra-low-temperature freezer, while the remaining plant material was used for biomass analysis. Samples stored in the ultrafreezer were ground under liquid nitrogen and returned to −80 °C for future analyses. Biochemical analyses performed on these samples included quantification of sucrose, starch, total soluble sugars (TSS), reducing sugars (RS), proline, free amino acids, total proteins, superoxide dismutase (SOD), hydrogen peroxide (H_2_O_2_), malondialdehyde (MDA), catalase (CAT), and peroxidase (POD).

### 4.4. Biomass

Fresh and dry mass analyses were performed under both water-stress and rehydration conditions to assess the impact of water deficit on plant development and their recovery capacity.

Fresh weight (FW), which indicates the water status of both shoots and roots, was measured immediately after collection using a precision balance and expressed in grams (g). For roots, samples were carefully cleaned to remove excess sand and then blotted with paper towels for 30 min to eliminate residual surface water.

Dry weight (DW), which excludes the influence of water and thus enables a precise assessment of plant growth [64,67], was determined for both shoots and roots. Samples were oven-dried with air circulation at 65 °C for 96 h until constant weight was achieved. After complete drying, the samples were weighed, and the values were expressed in grams (g).

Additionally, leaf water content (LWC) was calculated, serving as an important indicator of plant water status, particularly under water deficit conditions. This parameter provides the percentage of water in leaves relative to their total weight, reflecting the plant’s response to water deficit. LWC was calculated according to the formula:

(2) $$\mathrm{LWC}\;(\%)\;=\;\frac{FW-\;DW}{FW}\;\;\times \;100$$

where *FW* = fresh weight of leaves and *DW* = dry weight of leaves.

Total root length (RL) was measured to evaluate root system development. The roots were cleaned, and their lengths were measured manually using a measuring tape, with values expressed in centimeters (cm).

Plant height (PH) was determined by measuring the distance from the base to the highest point of the plant using a measuring tape. Values were expressed in centimeters (cm), providing an estimate of shoot growth in response to the different treatments.

At 21 days, after the rehydration phase, chlorophyll *a* fluorescence measurements were performed on plant leaves using the MINI-PAM-II photosynthetic yield analyzer. Measurements were taken between 8:00 and 10:00 a.m., an optimal time to minimize significant environmental fluctuations, such as changes in light intensity. Since no fluorescence measurements were conducted during the stress period, the data obtained at this stage reflect the physiological state of plants after recovery, i.e., following rehydration.

Measurements were carried out using metal clips (DLC-8) connected to the MINI-PAM sensor, placed in the middle region of leaves, on one side of the lamina, avoiding the central vein. Before each measurement, leaf portions were dark-adapted for 30 min to ensure that all reaction centers in that region reached the “open” state (non-reduced photochemical quencher). Data collected by the MINI-PAM were processed and calculated as described in Table 2 [68].

### 4.5. Chlorophyll, Macromolecules, H_2_O_2_ Content, and MDA

From the macerated samples stored in the ultrafreezer, 0.05 g of fresh leaf tissue was weighed and transferred to microtubes for extraction with ethanol. For each test tube, 350 µL of 100% ethanol was added, and the samples were heated in a water bath at 70–75 °C for 20 min. After this period, the samples were centrifuged at 14,000× *g* and 4 °C for 5 min. The supernatant obtained was carefully removed, and two subsequent extractions were performed with 80% and 50% ethanol, respectively, following the same procedure described above. The supernatants from all extractions (100%, 80%, and 50% ethanolic extracts) were stored at −20 °C for further analyses. All experimental protocols and techniques employed followed the methods established by López-Hidalgo et al. [69].

For pigment quantification, 25 µL of the ethanolic extract was pipetted into microplates, to which 145 µL of 100% ethanol was added for the analysis of chlorophyll *a*, chlorophyll *b*, and carotenoids. Absorbance measurements were taken at the following wavelengths: 647 nm for chlorophyll *a*, 623 nm for chlorophyll *b*, and 450 nm for carotenoids. Final pigment concentration values were determined from the absorbance readings and the fresh weight of the leaf tissues used in the extraction. Total chlorophyll content was obtained by summing chlorophyll *a* and chlorophyll *b* concentrations.

The quantification of malondialdehyde (MDA), an indicator of lipid peroxidation, was performed to assess oxidative stress in plant cells. For this, 250 µL of the ethanolic extract was combined with 250 µL of a solution containing 20% trichloroacetic acid and 0.5% thiobarbituric acid. The mixture was heated in a water bath at 95 °C for 30 min to promote the reaction between MDA and thiobarbituric acid. After this period, the samples were cooled on ice and centrifuged at 3000× *g* for 10 min at 4 °C to separate the solids. Following centrifugation, 150 µL of the supernatant was transferred in duplicate to microplates, and absorbance readings were taken at 532 nm and 600 nm. The MDA concentration was calculated based on the obtained absorbance values, using the methodology described by Heath and Packer [70]. The following equation gives the formula for calculating MDA concentration:

(3) $$\mathrm{MDA}\;=\;\frac{A533-A600}{\xi \times b}\;\;$$

where ξ is the molar extinction coefficient (1.56 × 10^−5^ cm^−1^) and b is the optical path length (1 cm). The MDA content was expressed in nmol (MDA) g^−1^ fresh weight.

The content of reducing sugars (RS) was determined using the DNS method, as described by Miller [71]. For this, 50 µL of ethanolic extract, 100 µL of distilled water, and 100 µL of DNS solution were added to microtubes. The tubes were then heated in a water bath at 100 °C for 5 min. After cooling, the volume was adjusted to 1000 µL, and absorbance readings were performed in duplicate at 620 nm.

The total soluble solids (TSS) content was quantified using the anthrone method [72]. For this analysis, 10 µL of ethanolic extract was mixed with 320 µL of distilled water and 670 µL of anthrone solution in microtubes. The tubes were heated in a water bath at 100 °C for 3 min. After cooling, absorbance readings were taken in duplicate at 620 nm.

Sucrose extraction was performed by adding 25 µL of ethanolic extract and 25 µL of 30% KOH solution to a microtube, which was then heated in a water bath at 40 °C for 15 min. Next, 50 µL of the obtained extract was mixed with 280 µL of distilled water and 670 µL of anthrone solution, and the mixture was placed in a water bath at 100 °C for 3 min to determine the sucrose content. After cooling, duplicate absorbance readings were taken at 620 nm.

For the quantification of total free amino acids, 50 µL of sodium citrate buffer, 50 µL of ethanolic extract, and 100 µL of ninhydrin solution (1% in 70% ethanol) were combined in a microtube. The samples were incubated in a water bath at 95 °C for 20 min. After cooling, absorbance was measured at 550/570 nm, allowing the quantification of free amino acids. The determination of proline was carried out using the same procedure, but with a reaction solution composed of 1% ninhydrin in 60% acetic acid and 20% ethanol. The samples were incubated at 95 °C for 20 min, cooled, and centrifuged at 2500× *g* for 1 min. Absorbance was measured at 520 nm to assess proline concentration.

Starch extraction was performed in the same microtube (containing both supernatant and pellet) after protein quantification. Initially, 70 µL of 1 M acetic acid was added and mixed, followed by 100 µL of a degradation solution composed of amyloglucosidase and 200 mM potassium acetate buffer (adjusted to pH 4.8). The samples were incubated in a water bath at 40 °C for 120 min. After this period, starch quantification was performed using the anthrone method [72], ensuring accurate measurement of starch content in the samples.

Additionally, the hydrogen peroxide (H_2_O_2_) content in the ethanolic extract was determined by reacting H_2_O_2_ with potassium iodide, and the absorbance was measured at 390 nm. H_2_O_2_ is another important marker of oxidative stress, and its quantification provides insights into the damage caused by stress in plants [73].

### 4.6. Antioxidant Activities

The extraction of antioxidant system enzymes, superoxide dismutase (SOD), peroxidase (POD), and catalase (CAT), involved the maceration of 0.20 g of fresh mass in liquid nitrogen, followed by the addition of 1.5 mL of a buffered solution (0.1 mol L^−1^ potassium phosphate pH 7.8, 0.1 mol L^−1^ EDTA pH 7.0, 0.5 mol L^−1^ dithiothreitol, 0.1 mol L^−1^ phenylmethylsulfonyl fluoride, 1 mmol L^−1^ ascorbic acid, and 0.022 g of polyvinylpolypyrrolidone). The supernatant was collected after centrifugation at 13,000× *g* for 10 min at 4 °C [74]. The supernatant was then collected for analysis in a microplate spectrophotometer (Epoch, BioTek, Seattle, WA, USA), according to the following methodologies: Havir and McHale [75]; Nakano and Asada [76], and Giannopolitis and Ries [77], respectively, for SOD, POD, and CAT.

### 4.7. Statistical Analyses

All statistical analyses were performed using R software, version 4.5.0 [78], through the specific packages tidyverse [79], readxl [80], ExpDes.pt [81], plotrix [82], corrplot [83], factoextra [84], FactoMineR [85], AgroR [86], and ggpattern [87].

The basic assumptions of the analysis of variance (normality, homoscedasticity, additivity, and independence of residuals) were tested. When normality was not met, a square root transformation (√x) was applied. Upon reaching significance in the F-test (*p* < 0.05), the treatment means of the measured variables were grouped using the Scott–Knott test (*p* < 0.05). The principal component analysis (PCA) was performed to assess the relationships among agronomic variables under water deficit and after rehydration, to identify which variables are most closely associated with the effects of niobium treatments under water stress conditions and subsequent rehydration, as well as to summarize the variability within a complex set of physiological and morphological measurements.

## 5. Conclusions

The use of hydropriming solutions containing ammonium niobate (V) oxalate (C_4_H_4_NNbO_9_) promoted a biostimulant effect during maize seed germination. However, under conditions of water deficit and rehydration, the application of niobium (Nb) may be associated with toxic effects, as it contributes to the reduction in shoot and root biomass, reflecting physiological limitations. Biochemical analysis showed that the presence of Nb worsened oxidative stress under water-deficit conditions. Seeds primed with this element produced seedlings with low efficiency in controlling lipid peroxidation, as indicated by elevated malondialdehyde (MDA) levels. Even after rehydration, MDA levels remained high, along with increased levels of hydrogen peroxide (H_2_O_2_). This result suggests activation of antioxidant defense mechanisms and cellular signaling, with increased activity of superoxide dismutase (SOD) and catalase (CAT), decreased peroxidase (POD) activity, and osmotic adjustments indicated by the accumulation of proline, total soluble sugars, and sucrose.

Additionally, Nb seed priming influenced photosynthetic performance in a treatment-dependent manner. Hydropriming with Nb exclusively induced phototoxic effects, whereas the addition of phosphate buffer partially mitigated these effects, preserving photosynthetic activity and cellular integrity. Overall, there was potential to influence the photosynthetic response of plants under water deficit; however, this also indicated signs of photoinhibition and decreased activation of energy dissipation mechanisms, pointing to phototoxic effects. Therefore, it appears that the behavior of Nb is strongly influenced by the priming solution, making it essential to consider its interaction with chemical buffers and the abiotic environment. To better understand the effects of this economically important element for Brazil, additional studies should be conducted throughout the entire crop cycle. These studies should focus on the biochemical and physiological mechanisms of Nb action and its impact on grain yield. Gaining knowledge of these interactions will be essential for developing effective seed conditioning strategies to mitigate abiotic stresses.

## Figures and Tables

**Table 1:** Description of the treatments used in the experiment.

Treatment	Hydropriming	Composition	Stress Condition	pH	Electrical Conductivity
T1	Distilled Water	H_2_O	Irrigation	5.142	72.0 µS cm^−1^
T2	Phosphate Buffer	0.5 mL K_2_HPO_4_ (1 M) + 0.5 mL KH_2_PO_4_ (1 M)	Irrigation	7.046	6599.3 µS cm^−1^
T3	Distilled Water	H_2_O	Water Deficit	5.142	72.0 µS cm^−1^
T4	Phosphate Buffer	0.5 mL K_2_HPO_4_ (1 M) + 0.5 mL KH_2_PO_4_ (1 M)	Water Deficit	7.046	6599.3 µS cm^−1^
T5	Ammonium Oxalate	13.45 mL (NH_4_)_2_C_2_O_4_·H_2_O (0.2 M), equivalent to 250 mg kg^−1^ ammonium niobate (V) oxalate (C_4_H_4_NNbO_9_)	Water Deficit	5.444	16,004.9 µS cm^−1^
T6	Ammonium Oxalate + Phosphate Buffer	13.45 mL of (NH_4_)_2_C_2_O_4_·H_2_O (0.2 M), in the same proportion as the concentration of 250 mg kg^−1^ of ammonium niobate (V) oxalate (C_4_H_4_NNbO_9_) + 0.5 mL of K_2_HPO_4_ (1 M) + 0.5 mL of KH_2_PO_4_ (1 M)	Water Deficit	6.593	18,079.9 µS cm^−1^
T7	Ammonium Niobate (V) Oxalate	250 mg kg^−1^ of ammonium niobate (V) oxalate (C_4_H_4_NNbO_9_), 1.35 mL of C_4_H_4_NNbO_9_ (1 M)	Water Deficit	3.571	1860.9 µS cm^−1^
T8	Ammonium Niobate (V) Oxalate + Phosphate Buffer	250 mg kg^−1^ of ammonium niobate (V) oxalate (C_4_H_4_NNbO_9_), 1.35 mL of C_4_H_4_NNbO_9_ (1 M) + 0.5 mL of K_2_HPO_4_ (1 M) + 0.5 mL of KH_2_PO_4_ (1 M)	Water Deficit	6.247	70,641.1 µS cm^−1^

The concentration of 250 mg kg^−1^ of niobium (Nb), as ammonium niobate (V) oxalate (C_4_H_4_NNbO_9_), was selected for seed priming based on preliminary dose–response experiments, which demonstrated that this concentration enhanced maize seed germination and early seedling development without inducing toxic effects, whereas higher concentrations resulted in toxicity and lower doses showed negligible physiological responses.

**Table 2:** Analyses performed using MINI-PAM.

Parameter	Description	Formula
PAR	Photosynthetically active radiation. Value measured directly by MINI-PAM and used in the calculation of ETR	-
F_O_	Minimum Chl *a* fluorescence yield in the dark-adapted state	-
F_M_	Maximum Chl *a* fluorescence yield in the dark-adapted state	-
F_V_/F_M_	A quantity related to the maximum quantum yield of PSII photochemistry	$F_{V}/F_{M}=\frac{FM-FO}{FM}$
F_M_′	Maximum fluorescence (measured during a saturation pulse under growth light conditions)	-
F	Fluorescence before the saturation pulse	-
Yield PSII (ΦPSII)	Effective quantum yield of PSII	$\mathrm{Yield}=\frac{FM\prime -F}{FM\prime }$
Y(NO)	Quantum yield of non-regulated energy dissipation	$Y(\mathrm{NO})=\frac{F}{FM}$
Y(NPQ)	Quantum yield of regulated energy dissipation via NPQ-dependent mechanisms	Y(NPQ) = 1 − (YIELD − YNO)
F_O_′	Minimum fluorescence under light	-
qP	Photochemical quenching coefficient based on the ratio between open and closed centers	$\mathrm{qP}=\frac{FM\prime -F}{FM-FO}$
qL	Photochemical quenching coefficient based on the fraction of open PSII reaction centers	$\mathrm{qL}=\mathrm{qP}\;\times \;\frac{FO}{F}$
qN	Non-photochemical quenching coefficient	$\mathrm{qN}=\frac{FM-FM\prime }{FM-FO}$
NPQ	Stern–Volmer non-photochemical quenching	$\mathrm{NPQ}=\frac{FM-FM\prime }{FM\prime }$
ETR	Electron transport rate	ETR = YIELD × PAR × 0.5 × ETR factor

## Data Availability

The data supporting this study’s findings are available on request from the corresponding author. The data are not publicly available due to all relevant data being included within the article; therefore, no additional raw data is available for sharing.
